# *In Vivo* Calcium Imaging of CA3 Pyramidal Neuron Populations in Adult Mouse Hippocampus

**DOI:** 10.1523/ENEURO.0023-21.2021

**Published:** 2021-08-21

**Authors:** Gwendolin Schoenfeld, Stefano Carta, Peter Rupprecht, Aslı Ayaz, Fritjof Helmchen

**Affiliations:** 1 Laboratory of Neural Circuit Dynamics, Brain Research Institute, University of Zurich, Zurich CH-8057, Switzerland; 2 Neuroscience Center Zurich, University of Zurich, Zurich CH-8057, Switzerland

**Keywords:** auto-associative network, calcium imaging, complex spike burst, hippocampus CA3, juxtacellular, locomotion

## Abstract

Neuronal population activity in the hippocampal CA3 subfield is implicated in cognitive brain functions such as memory processing and spatial navigation. However, because of its deep location in the brain, the CA3 area has been difficult to target with modern calcium imaging approaches. Here, we achieved chronic two-photon calcium imaging of CA3 pyramidal neurons with the red fluorescent calcium indicator R-CaMP1.07 in anesthetized and awake mice. We characterize CA3 neuronal activity at both the single-cell and population level and assess its stability across multiple imaging days. During both anesthesia and wakefulness, nearly all CA3 pyramidal neurons displayed calcium transients. Most of the calcium transients were consistent with a high incidence of bursts of action potentials (APs), based on calibration measurements using simultaneous juxtacellular recordings and calcium imaging. In awake mice, we found state-dependent differences with striking large and prolonged calcium transients during locomotion. We estimate that trains of >30 APs over 3 s underlie these salient events. Their abundance in particular subsets of neurons was relatively stable across days. At the population level, we found that co-activity within the CA3 network was above chance level and that co-active neuron pairs maintained their correlated activity over days. Our results corroborate the notion of state-dependent spatiotemporal activity patterns in the recurrent network of CA3 and demonstrate that at least some features of population activity, namely co-activity of cell pairs and likelihood to engage in prolonged high activity, are maintained over days.

## Significance Statement

*In vivo* measurements of neuronal population activity may reveal how the mammalian hippocampus supports fundamental brain functions such as memory. So far, however, calcium imaging in deep hippocampal regions such as the CA3 subfield has been rarely achieved. Here, we use a red calcium indicator to measure CA3 pyramidal neuron activity in the mouse brain during different states [anesthetized vs awake (resting or running)] and across days. Most CA3 pyramidal neurons displayed calcium transients consistent with complex spike bursts. During running, salient large and prolonged calcium signals were prominent. Some features of neuronal activity remained relatively stable over days, e.g., co-activity in neuronal pairs. Our study further expands CA3 calcium imaging in behaving mice, fostering analysis of CA3 network activity.

## Introduction

Neuronal populations in the hippocampal CA3 subfield are part of the mammalian brain circuit that is essential for spatial navigation, memory formation, and cognition (Kesner, 2007; Hartley et al., 2013; Rolls, 2016; Rebola et al., 2017; Hainmueller and Bartos, 2018). CA3 pyramidal neurons are special in forming an auto-associative recurrent network enabling memory encoding and pattern completion (Rolls, 2007; Kesner and Rolls, 2015; Guzman et al., 2016; Knierim and Neunuebel, 2016). The functional properties of CA3 pyramidal neurons have been characterized largely with electrophysiology, using extracellular recordings (Fox and Ranck, 1975; Csicsvari et al., 2000; Henze et al., 2002; Leutgeb et al., 2004; Frerking et al., 2005; Mizuseki et al., 2012; Oliva et al., 2016), *in vivo* intracellular and juxtacellular recordings (Epsztein et al., 2011; Kowalski et al., 2016; Zucca et al., 2017; Diamantaki et al., 2018; Hunt et al., 2018; Malezieux et al., 2020), and whole-cell recordings in brain slices (Jonas et al., 1993; Hemond et al., 2008; Hunt et al., 2018; Raus Balind et al., 2019). Pyramidal neurons in CA3 show properties distinct from CA1 (Mizuseki et al., 2012; Oliva et al., 2016) but display heterogeneity within their population (Hunt et al., 2018; Cembrowski and Spruston, 2019; Ding et al., 2020). For CA3 pyramidal neurons, mean firing rates typically range from 0.3–5 Hz *in vivo* (Henze et al., 2002; Wittner and Miles, 2007; Mizuseki et al., 2012; Kowalski et al., 2016; Oliva et al., 2016; Ding et al., 2020), lower than for CA1 pyramidal neurons but higher when compared with dentate gyrus (DG) granule cells. As a prominent feature, hippocampal pyramidal neurons, especially in CA3, exhibit bursts of action potentials (APs) with interspike intervals (ISIs) <6 ms (Fox and Ranck, 1975; Frerking et al., 2005; Mizuseki et al., 2012; Kowalski et al., 2016; Oliva et al., 2016; Raus Balind et al., 2019). These complex spike bursts involve regenerative dendritic mechanisms and have been implicated in activity-dependent plasticity (Lee et al., 2012; Grienberger et al., 2014; Bittner et al., 2015, 2017; Diamantaki et al., 2018; Raus Balind et al., 2019). They are also associated with network synchronization events in CA3 (Miles and Wong, 1983; Menendez De La Prida et al., 2006; Wittner and Miles, 2007; Marissal et al., 2012), especially sharp-wave ripples (Buzsáki, 1986; Csicsvari et al., 2000; Harris et al., 2003; Hunt et al., 2018).

Despite these advances in electrophysiological studies, our understanding of CA3 network dynamics and its computational roles remains limited. Optophysiology offers promising complementary approaches, especially in terms of longitudinal imaging of the same neuronal population. However, because of the difficulties in accessing deeper brain regions, hippocampal imaging studies have lagged behind similar studies in neocortex. Only during the last decade, *in vivo* calcium imaging in hippocampus became possible, typically by removing the overlying cortical tissue and using either two-photon microscopy in head-fixed animals (Dombeck et al., 2010; Grienberger et al., 2014; Hainmueller and Bartos, 2018; Kinsky et al., 2018) or mini-endoscopes in freely-moving mice (Ziv et al., 2013; Rubin et al., 2015; Gonzalez et al., 2019; Stefanini et al., 2020). While initial studies mainly targeted CA1 as the most accessible region, only at a later stage chronic and functional imaging was also established in the DG (Pilz et al., 2016, 2018; Danielson et al., 2017; Hainmueller and Bartos, 2018; Stefanini et al., 2020). In our own previous study (Pilz et al., 2016), by applying GCaMP6 and specifically R-CaMP1.07, a red calcium indicator that facilitates deep imaging (Ohkura et al., 2012; Bethge et al., 2017), we confirmed sparse activity of DG granule cells and described its variation across behavioral states. Functional imaging in CA3 is as challenging as in DG and therefore has been achieved in only few studies until today (Rajasethupathy et al., 2015; Hainmueller and Bartos, 2018; Rashid et al., 2020). As an emerging field, CA3 imaging provides new opportunities to address key questions about cellular and circuit mechanisms of neural coding and plasticity in this region.

Here, we establish *in vivo* calcium imaging of CA3 pyramidal neurons using an approach similar to our previous DG study (Pilz et al., 2016). We characterize basic features of CA3 calcium transients and calibrate them in terms of underlying APs using simultaneous juxtacellular recordings. We find heterogeneous CA3 activity patterns across behavioral states and discover particularly prominent prolonged calcium transients that occur in neuronal subsets during running. Moreover, our longitudinal imaging results indicate that CA3 population activity at least partially remains stable across days, particularly with respect to the co-activity of neurons within sub-ensembles.

## Materials and Methods

### Animals and R-CaMP1.07 labeling

All experimental procedures were conducted in accordance with the ethical principles and guidelines for animal experiments of the Veterinary Office of Switzerland and were approved by the Cantonal Veterinary Office in Zurich. For the experiments, male and female mice with a Tg(Grik4-cre)G32-4Stl background were used (*n* = 6). These mice show a dense expression of Cre-recombinase rather specific to CA3 hippocampal pyramidal neurons (MGI:2387441; Nakazawa et al., 2002). We induced expression of the red fluorescent calcium indicator R-CaMP1.07 (Ohkura et al., 2012) in CA3 pyramidal neurons by stereotaxic injection of AAV1-EFα1-DIO-R-CaMP1.07 in six- to nine-week-old adult mice (coordinates: AP −2, ML +1.8, DV −2.2; in mm from bregma; 300 nl with a virus titer of ∼1 × 10^7^ vg/nl).

### Hippocampal window implantation

**Figure 1.**
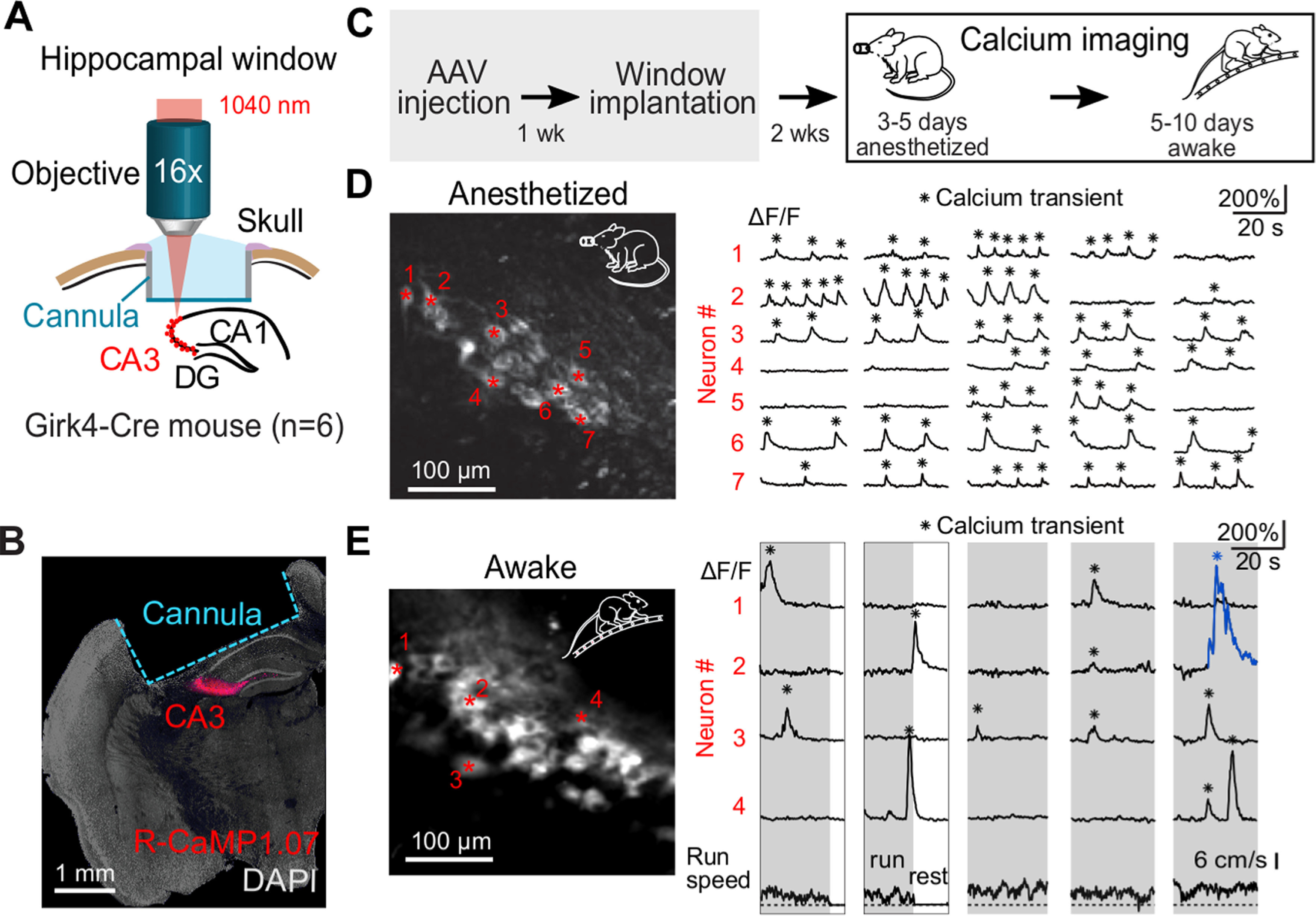
*In vivo* two-photon imaging of CA3 pyramidal neuron populations during wakefulness and anesthesia. ***A***, Schematic illustration of experimental setup showing the chronic window implant above the corpus callosum and area CA1 of the intact hippocampus for R-CaMP1.07 calcium imaging in CA3. ***B***, Histologic coronal cross-section of the fixed brain after *in vivo* imaging sessions showing R-CaMP1.07-labeled CA3 pyramidal neurons and the hippocampal window implant. ***C***, Experimental time line. After recovery from the surgery, three consecutive days of anesthetized imaging were conducted. This was followed by 6–10 d of awake imaging including at least three consecutive imaging days. ***D***, Example FOV of an anesthetized two-photon imaging session. Example neurons are labeled by red asterisks and their respective ΔF/F traces are displayed on the right. Detected calcium transient peaks are labeled with black asterisks. ***E***, Example FOV and ΔF/F traces of neurons recorded during wakefulness. An example large calcium transient is labeled in blue. Run speed of the animal is shown below.

Chronic access for CA3 imaging was obtained by the implantation of a hippocampal window (Pilz et al., 2016). One week after the virus injection, we performed a 3-mm diameter craniotomy centered at the injection site and implanted a stainless-steel cannula with a front glass window. After removing the bone, we gently aspirated the underlying cortical tissue until the corpus callosum fibers became visible. A stainless-steel cannula (Ø 3 mm, 1.5 mm length) covered by a glass coverslip (Ø 3 mm, 0.17-mm thickness) was inserted into the cavity and secured in place using dental acrylic cement (Ivoclar Vivadent; Fig. 1*A*,*B*). Additionally, an aluminum post for head fixation during imaging was attached to the skull. After a recovery period, mice were handled by the experimenter, habituated to head fixation, and accustomed to run on a ladder wheel (Ø 23 cm) with regularly spaced rungs (1-cm spacing) during head fixation. Approximately two weeks after the surgery, neuronal population activity was imaged under isoflurane anesthesia (1–2% in oxygen) on three to five consecutive days. The same neuronal populations that were imaged in the anesthetized condition were repeatedly imaged in awake animals for 5–10 d, of which at least 3 d were consecutive (Fig. 1*C*).

### Two-photon calcium imaging

We used a custom-built two-photon microscope based on the Sutter movable objective microscope (MOM) type, equipped with a water immersion 16× objective (CFI LWD 16×/0.80; Nikon), a Pockels cell (model 350/80 with controller model 302RM, Conoptics), and galvanometric scan mirrors (model 6210; Cambridge Technology), controlled by HelioScan software (Langer et al., 2013). R-CaMP1.07 was excited by ∼230-fs pulses at 80 MHz provided by a ytterbium-doped potassium gadolinium tungstate (Yb:KGW) laser (1040 nm; >2-W average power; model Ybix; Time-Bandwidth Products). Emitted fluorescence was detected by a photomultiplier tube after passing through a 610/75-nm bandpass filter (AHF Analysetechnik). Laser intensities during imaging were 56–78 mW under the objective.

In anesthetized experiments, mice were anesthetized with isoflurane (1–2% in O_2_). Body temperature was monitored continuously with a thermosensor and kept at 37°C with a heating blanket. For awake experiments, the head-fixed mouse was placed on the ladder wheel and was free to run. Running speed and running distance during calcium imaging were recorded at 40 Hz with a rotary encoder (Phidgets, 12V/0.2Kg-cm/230RPM 10:1 DC gear motor with encoder). The activity of R-CaMP1.07-expressing CA3 pyramidal cells was recorded in trials of 30-s duration, with 10-s inter-trial intervals (maximum of 30 trials per day). Recordings were performed in the distal part of CA3 (CA3a), which lays in the proximity of CA2. In all sessions, imaging across a field of view (FOV) of 325 × 325 μm^2^ was performed at 10-Hz frame rate.

### *In vivo* electrophysiology

Electrophysiological recordings, combined with *in vivo* calcium imaging, were performed in acute *in vivo* preparations of Tg(Grik4-cre)G32-4Stl expressing R-CaMP1.07 mice (*n* = 3; at least two weeks after injection). Mice were anesthetized with isoflurane and the temperature was maintained at 37°C. A stainless steel plate was fixed to the exposed skull using dental acrylic cement. A 4-mm diameter craniotomy was performed, centered above the virus injection locus. The overlying cortex was aspirated until the corpus callosum became visible. A 1%-agarose gel was filled into the cavity to reduce tissue motion. Juxtacellular recordings from R-CaMP1.07-expressing CA3 pyramidal neurons were obtained with glass pipettes (4- to 6-MΩ pipette resistance) filled with Ringer’s solution. To facilitate visually-guided targeting of individual neurons, the pipette was coated with BSA Alexa Fluor 594 (Invitrogen). APs were recorded juxtacellularly in current clamp mode at 10-kHz sampling rate using an Axoclamp 2B amplifier (Molecular Devices, Molecular Devices) and digitized using Clampex 10.2 software. Simultaneously, we performed two-photon calcium imaging at 20-Hz frame rate, zoomed-in on the recorded neuron to optimize signal-to-noise ratio.

### Perfusion and histology

After the last awake imaging session, mice were administered a lethal dose of pentobarbital (Ekonarcon, Streuli) and transcardially perfused with sterile NaCl (0.9%) followed by 4% paraformaldehyde (PFA; 0.1 M phosphate buffer, pH 7.4). We cut 40-μm coronal brain slices and acquired histologic images with a confocal laser-scanning microscope (Olympus FV1000) using 546-nm laser light for R-CaMP1.07 excitation (Fig. 1*B*).

### Data analysis

Electrophysiological data were analyzed using routines in IGOR (Wavemetrics). R-CaMP1.07 fluorescence signals were analyzed using custom-written macros in ImageJ (Schindelin et al., 2012) and MATLAB routines (The MathWorks). For motion correction of calcium imaging movies, we applied a hidden Markov model line-by-line motion correction algorithm (Dombeck et al., 2007). We excluded trials that obviously were insufficiently motion-corrected based on visual inspection. Regions of interest (ROIs) corresponding to individual neurons were manually selected from the mean fluorescence image of a single-trial time series. Background fluorescence was estimated as the bottom first percentile fluorescence signal across the entire session and subtracted before calculating the relative percentage fluorescence change from baseline ΔF/F = (F–F_0_)/F_0_. Baseline fluorescence F_0_ was computed as 51st percentile of the fluorescence signal in an 8-s sliding window. ΔF/F traces were smoothed with a five-point first-order Savitsky–Golay filter.

**Figure 2.**
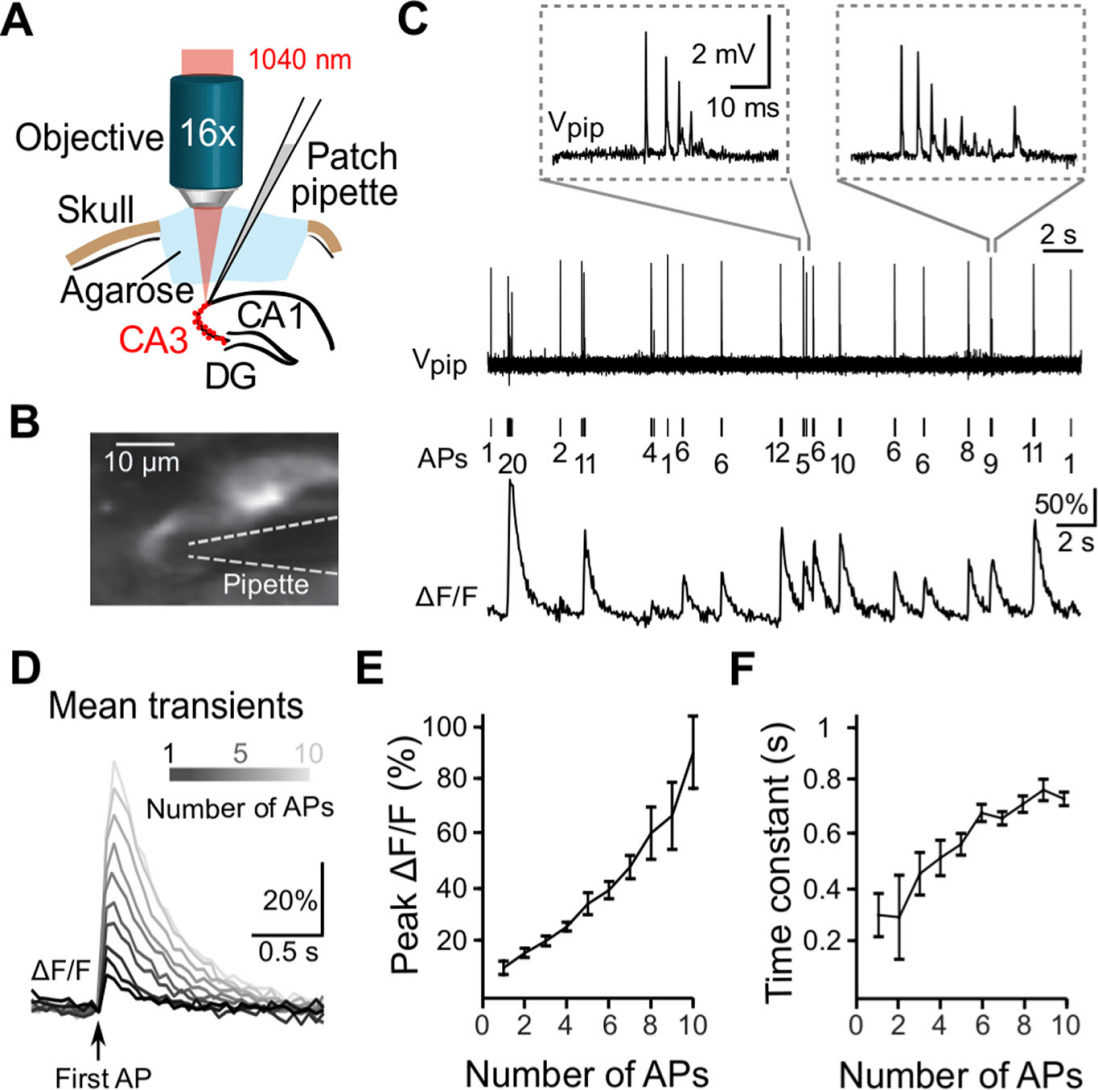
Calibration of *in vivo* spiking activity of CA3 pyramidal neurons using simultaneous two-photon calcium imaging and juxtacellular voltage recordings in acute experiments. ***A***, Schematic of experimental setup. ***B***, Maximum-intensity projection of two-photon images of R-CaMP1.07-labeled CA3 pyramidal neurons together with the recording pipette in juxtacellular configuration. ***C***, Example traces of simultaneously recorded calcium transients (ΔF/F) and spontaneous APs (V_pip_) during isoflurane-anesthesia. The number of APs per burst is indicated below. Insets show two example burst events magnified. ***D***, Average calcium transients caused by 1–10 APs (within 200 ms) aligned to the occurrence of the first AP. ***E***, Relationship between peak ΔF/F changes and number of APs (mean ± SEM). ***F***, Relationship between the calcium transient decay time constant and the number of APs (exponential fits; error bars indicate 90% confidence interval). The heterogeneity of burst events is further analyzed in Extended Data Figure 2-1.

For characterization of R-CaMP1.07 signals based on ground truth data (Fig. 2), we aligned the simultaneously recorded electrophysiological traces and fluorescence signals at the start of recording. We determined the peak amplitude of isolated calcium transients (i.e., with no spiking activity in a 2-s period before the first AP associated with the calcium transient) and counted the number of underlying APs. To focus on quasi-impulse-like responses, we only considered transients with APs that occurred within a time window of maximally 200-ms duration. For averaging, calcium transients were aligned to the first AP of a given event.

**Figure 3.**
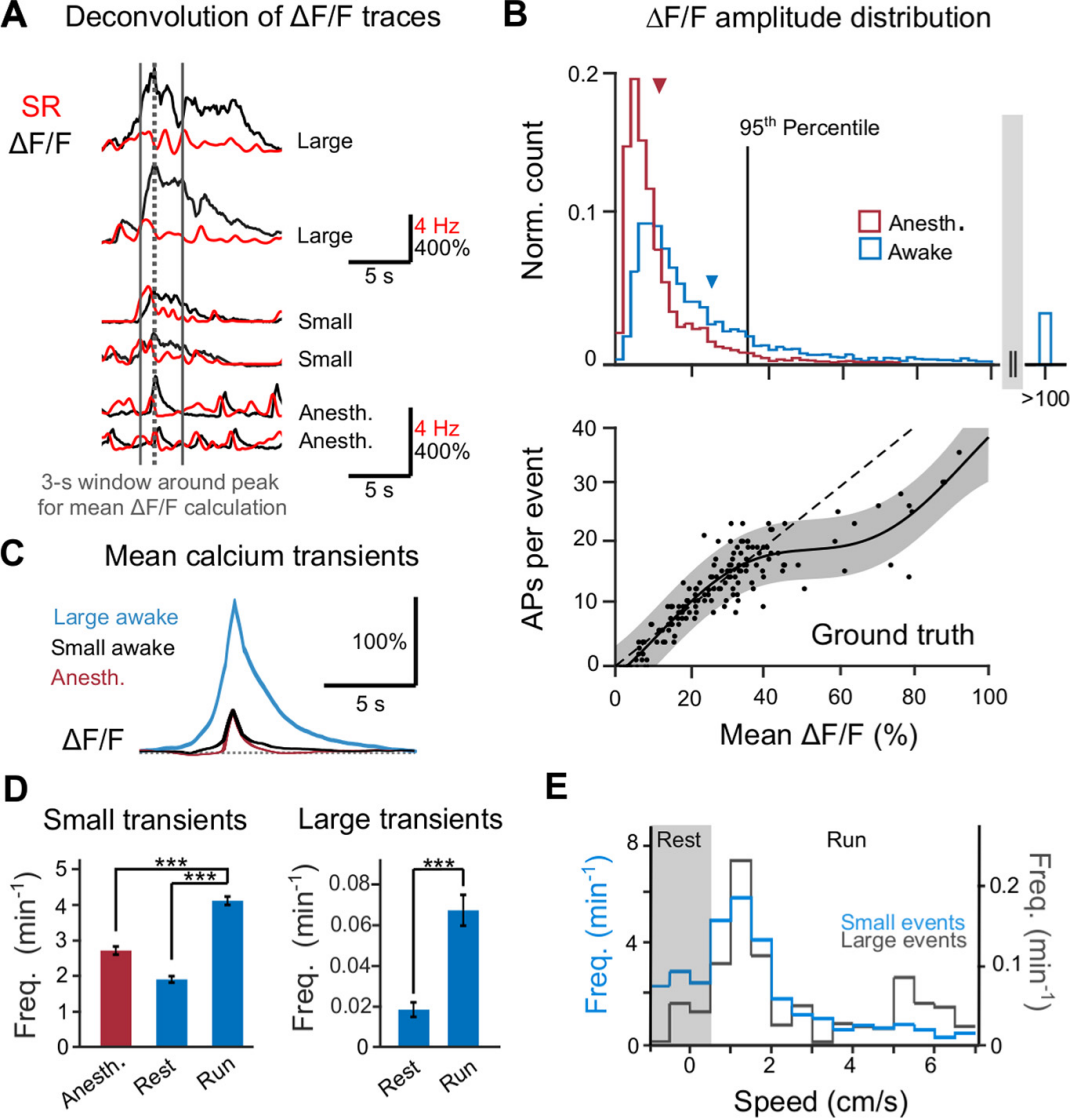
Large and prolonged calcium events in CA3 pyramidal neurons during wakefulness. ***A***, Example ΔF/F traces (black) with corresponding estimated spike rates (SR, red). ***B***, Distribution of mean ΔF/F level in a 3-s time window around calcium transient peaks (dashed line in ***A***) under anesthesia (red) and during wakefulness (blue). Wakeful transients that exceeded the 95th percentile of anesthetized mean ΔF/F levels (37% mean ΔF/F) were classified as large events. Arrows indicate the respective mean of each distribution (awake: 23 ± 24%, anesthetized: 11 ± 10%, mean ± SD, *p* < 0.001, two-sided Wilcoxon rank-sum test). The lower panel, as a calibration look-up table for the upper panel, shows the relation of mean ΔF/F level to the number of APs extracted from the ground-truth dataset. The linear reference line is fitted to data points with mean ΔF/F values <40%. The 95% confidence corridor (gray shading) is based on a model-free fit of all data points. ***C***, Average shape of calcium transients classified as anesthetized events, awake small and awake large events (mean ± SEM). ***D***, left, Mean frequency of small calcium transients per neuron and session under anesthetized, awake resting, and awake running conditions (mean ± SEM; resting vs anesthetized: 0.70 ± 0.04, running vs anesthetized: 1.53 ± 0.08, running vs resting: 2.19 ± 0.12; ratio of means ± error propagation of SEM). Right, Mean frequency of large calcium transients across neurons during awake resting and awake running condition (running vs resting: 3.67 ± 0.83; ratio of means ± error propagation of SEM); ***p <0.001, one-way ANOVA. ***E***, Frequency distribution of 1859 small awake events (blue) and 947 large awake events (black), normalized to the times spent at specific speeds, as a function of running speed.

10.1523/ENEURO.0023-21.2021.f2-1 Extended Data Figure 2-1 Heterogeneity of burst events in CA3 pyramidal neurons from cell-attached ground-truth recordings. ***A***, Electrophysiological recordings of three example bursts. Example two and three show intermittent bursts. ***B***, Overview of all recorded bursts with up to 17 APs. Amplitudes were normalized to the maximum AP amplitude within the burst. ***C***, Distribution of inter spike interval times pooled from all recordings (four neurons, three mice). The bimodal distribution can be split into two clusters using k-means clustering with one cluster around 5.3 ± 7.3 ms (mean ± SD) and the second cluster at 0.81 ± 1.51 s. ***D***, Mean ΔF/F transient and peri-event histogram of underlying spikes for all ΔF/F transients detected as in Figure 3 (see detection criteria in Materials and Methods). In addition, we show the mean instantaneous SR, estimated from the deconvolved ΔF/F traces. The SRs were temporally smoothed compared to the peri-event histogram since our method to estimate SRs was trained with temporally smoothed ground truth data in order to be more resilient against noise (Materials and Methods). Averages of 173 detected events; shaded corridors show SEM. Download Figure 2-1, EPS file.

We also used the ground truth dataset (*n* = 4 neurons from three mice; a total of 33 min of recording and 5025 APs) to train a supervised algorithm based on neural networks to deconvolve calcium transients and estimate the underlying spike rates (SRs). The deconvolution algorithm, which we present in detail in a separate paper (Rupprecht et al., 2021), was trained on the R-CaMP1.07 ground truth data, which were re-sampled to the 10-Hz frame rate used for awake imaging. The noise level of the ground truth data was adjusted to match the noise level of each neuron of the population imaging data by adding Poisson noise. Spike trains used to train the network were temporally filtered with a Gaussian [∼470-ms full-width-half-maximum (FWHM)]. Prediction of SRs using this approach is expected to show correlation values of 0.79 ± 0.16 (mean ± SD) with the ground truth data, thus explaining ∼60% of the variance (Rupprecht et al., 2021).

**Figure 4.**
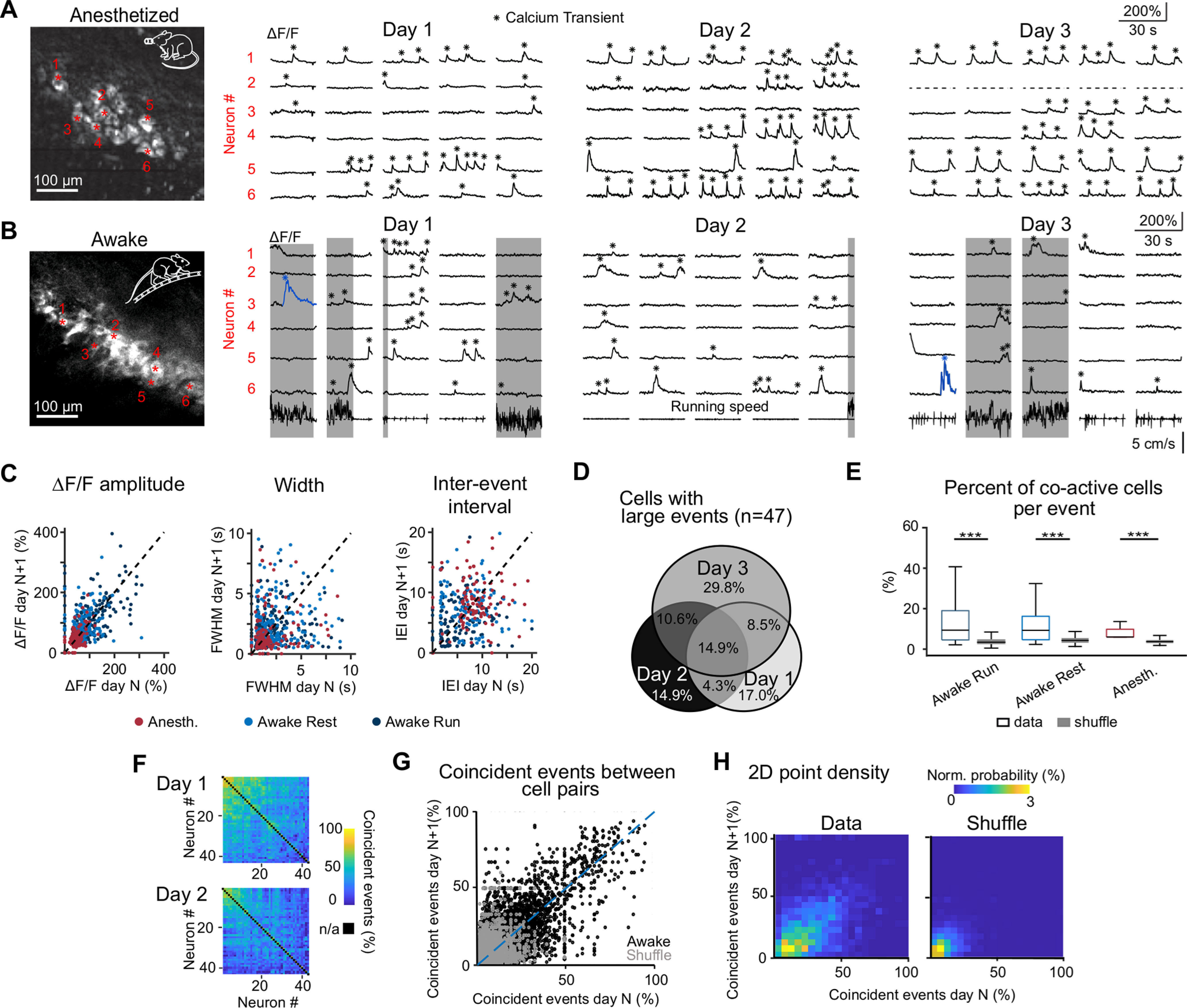
Longitudinal functional imaging of CA3 population activity over consecutive recording days. ***A***, Example FOV (left) and calcium transients of selected neurons over three consecutive imaging days during anesthesia. ***B***, Example FOV and across-days calcium transient examples during wakefulness. Example large calcium events are labeled blue. Periods of locomotion are highlighted in gray. ***C***, Comparison of mean ΔF/F transient amplitude, mean FWHM, and mean IEI per neuron over consecutive imaging days (dashed lines represent unity lines; for Pearson correlations see main text). For a plot of these calcium transient properties across all neurons and days, see Extended Data Figure 4-1. ***D***, Percentage of cells firing large events for each day and over consecutive days (number of neurons: *n*_day1_ = 21, *n*_day2_ = 21, *n*_day3_ = 30, *n*_day1+day2_ = 9, *n*_day2+day3_ = 12, *n*_day1+day3_ = 11, *n*_day1+day2+day3_ = 7). ***E***, Percentage of neurons in a FOV that show co-activity within a 1-s time window surrounding a detected event, compared with randomly shuffled event times (***p < 0.001; box displays median and 25th and 75th percentiles, whiskers indicate first and 99th percentiles; resting vs anesthetized 2.15 ± 0.05, running vs anesthetized 2.57 ± 0.06, resting vs running 1.20 ± 0.02; ratio of means ± error propagation of SEM). ***F***, Percentage of coincident events between all neuron pairs of one example FOV over 2 d. Hierarchical link clustering was performed on the co-activity matrix of day 1, and the resulting order of neurons was maintained for day 2. ***G***, Comparison of percentage of coincident events per neuron pair over consecutive imaging days. For this plot all data from all FOVs were pooled (experimental data: ρ = 0.34, shuffled data: ρ = 0.06). Dashed line represents unity line. ***H***, Normalized 2D density plot based on ***G***. For temporal aspects of co-activity and its dependence on neuron pair distance see Extended Data Figures 4-2, 4-3, respectively.

For analysis of population imaging data in Figures 3, 4, we defined detectable calcium transients as fluorescence signals that deviated from baseline by >3 SD for anesthetized imaging and >4 SD for awake conditions. We applied the more stringent criterion for awake conditions because of increased noise levels and possible motion artifacts during wakefulness. For every threshold-crossing event we determined the calcium transient peak as the first maximum found by the MATLAB function *findpeaks* (using minimal peak prominence of 20% ΔF/F and minimal peak separation of 1.5 s). We then excised 3-s segments around the detected calcium transient events (−1 to +2 s relative to the peak) and estimated the underlying SRs using the deconvolution algorithm. For each event, we computed the mean ΔF/F value in the 3-s time window, reflecting the integral cellular activity causing the calcium transient. For the awake recordings, we defined “large” calcium transients as those that displayed mean ΔF/F values larger than the 95th percentile of the distribution of mean ΔF/F values for all transients recorded during anesthesia. The ground truth data recorded during anesthesia did not fully cover calcium transient amplitudes and shapes representative of the large and prolonged calcium transients observed during wakefulness. To estimate the number of APs during these calcium transients in Figure 3, we therefore used a model-free look-up table based on the integral of the SR predictions in the excised 3-s calcium transient segment (Fig. 3*B*, bottom). A 95% confidence corridor for the data was obtained by Gaussian process regression (with the MATLAB function *fitrgp*), using squared exponentials as the kernel functions and optimizing hyperparameters of the Gaussian process regression with cross-validation. For extra analysis based on the deconvolved calcium transients, we detected peaks of the estimated SR trace with a similar procedure as for ΔF/F traces (minimal peak prominence 1.5 Hz, minimal peak separation 1.5 s).

10.1523/ENEURO.0023-21.2021.f4-1 Extended Data Figure 4-1 Properties of calcium transients of CA3 neurons over consecutive imaging days. Neuron-wise analysis of (***A***) mean ΔF/F amplitude, (***B***) mean IEI, and (***C***) mean transient FWHM calculated over one session for each neuron on three consecutive days in the anesthetized, awake resting and awake running condition. Neurons that were recorded on all 3 d were sorted according to their properties on the first imaging day (*n*_anesth._ = 91 cells, *n*_awake_ = 181 cells, 6 mice, 6 FOVs imaged under anaesthesia, 10 FOVs imaged during wakefulness). Download Figure 4-1, EPS file.

10.1523/ENEURO.0023-21.2021.f4-2 Extended Data Figure 4-2 Temporal aspects of neuronal co-activity in CA3. ***A***, Effect of window size on the Pearson’s correlation measure of stability of co-activity across days (compare Fig. 4*G*). The analysis was performed with ΔF/F traces in the awake and anesthetized condition as well as with estimated SRs in the awake condition (SR). For the 0.1-s window, the analysis was performed on the single imaging frame for which the transient peak of the event was detected. Windows up to a duration of 2 s started 0.5 s before the transient peak. From a window size of 3 s onwards, windows started 1 s before the transient peak. Dashed vertical line indicates the1-s window chosen for analysis in the main text. ***B***, Average temporal profile of ΔF/F traces (top) and estimated SR (bottom) in co-active neurons aligned to the peak of detected events in reference neurons. Mean traces (±SEM) for events co-active to large events are shown in red and events co-active to small events in blue. Note that the average traces do not include the original detected events (as in Fig. 3*C*) but only calcium transients in co-active neurons. ***C***, Peri-event histograms of the calcium transient peaks for small (top) and large (bottom) events in co-active neurons, aligned to the calcium transient peak of the originally detected reference event. Download Figure 4-2, EPS file.

10.1523/ENEURO.0023-21.2021.f4-3 Extended Data Figure 4-3 Influence of physical Euclidean distance between neurons on neuron-pair co-activity. ***A***, left, Correlation between ΔF/F traces of neuron pairs (*y*-axis) as a function of the Euclidian distance between neuronal ROI centroids (*x*-axis). Right, The values for neighboring neuron pairs (distances ≤10 μm) show higher mean correlation values than more distant neuron pairs (0.30 ± 0.28 and 0.14 ± 0.30; mean ± SD). ***B***, Pearson correlation values of neuron-pair co-activity across multiple days for variable temporal windows (windows defined as in Extended Data Fig. 4-2*A*) from all neuron pairs (black) and from neuron pairs separated by more than 10 μm (orange), showing similar results. Dashed vertical line indicates the 1-s window chosen for analysis in the main text.1 Download Figure 4-3, EPS file.

For analysis of neuronal population activity under anesthetized, awake resting, and awake running conditions, we identified in each FOV the neurons that were co-active with the events detected in a reference neuron. Co-activity was defined as showing a calcium transient peak within a 1-s window surrounding the peak of the reference event (0.5 s before to 0.5 s after). The percentage of coactive neurons per event of all neurons analyzed in the FOV was determined separately for anesthetized and awake conditions. Results were compared with shuffled co-activity values obtained by randomizing the peak times of detected calcium transients in tested neurons (taking the mean of 100 randomizations). To evaluate the statistical difference between conditions we subtracted the mean values obtained from shuffled data and then computed the ratio between two conditions, e.g., awake resting versus running. We estimated the SEM by Gaussian error propagation.

Additionally, we assessed the stability of co-activity of neuron pairs over multiple days. For each day, for which a pair of neurons (e.g., neurons A and B) was recorded, we determined the fraction of co-active events compared with all events in these neurons. Co-activity again was -defined by the co-occurrence of an event in neuron B within a 1-s window around a detected event in reference neuron A and vice versa for reference events in neuron B (resulting in a symmetric co-activity measure by taking the mean). For the shuffle control, we randomized peak times of all events in the non-reference neurons (100 times repeated per neuron pair). Stability was assessed by the Pearson’s correlation coefficient (ρ) comparing co-activity values on 1 d (day *N* + 1) with the previous day (*N*). We also repeated the co-activity analysis for the deconvolved calcium transients (SR traces), which did not change the results. To probe the robustness of the results against the choice of the time window, we tested additional time windows from 0.1- to 4.5-s duration (Extended Data Fig. 4-2*A*). To analyze the dependence of co-activity values on the distance between neuron pairs, we used the Euclidean distances between the centroids of the respective ROIs (Extended Data Fig. 4-3).

### Run speed analysis

We down-sampled running speed to the 10-Hz imaging frame rate and defined periods with >0.5 cm/s speed as “run” periods and periods with lower speeds as “rest” periods. The numbers of small and large calcium transients per minute during wakeful resting or locomotion were determined and distributions were compared using one-way ANOVA. The normalized frequency distributions of wakeful small and large events across run speeds were compared using a Kolmogorov–Smirnov test.

## Results

### *In vivo* two-photon calcium imaging of CA3 pyramidal neurons

We established *in vivo* two-photon imaging of CA3 neuronal population activity through a chronically implanted window after removal of cortical tissue overlying the hippocampus (Fig. 1*A*,*B*). To induce expression of a genetically encoded calcium indicator specifically in CA3 pyramidal neurons, we injected Grik4-Cre transgenic mice with a virus driving Cre-dependent expression of the red-shifted calcium indicator R-CaMP1.07 (Ohkura et al., 2012; Bethge et al., 2017; Fig. 1*B*). Following chronic window preparation and habituation of the mouse to head-fixation, we performed calcium imaging of R-CaMP1.07-expressing CA3 pyramidal neurons in several sessions of around 30 min in duration, spread over consecutive days and under either anesthetized or awake condition (Fig. 1*C*). During awake recordings, mice were free to run or rest on a ladder wheel placed under the two-photon microscope. We continually measured running speed and used a threshold to distinguish behavioral states by defining run and rest periods.

Nearly all neurons exhibited calcium transients indicative of neuronal spiking activity in both anesthetized and awake condition (Fig. 1*D*,*E*). For every mouse (*n* = 6) >90% of cells showed at least one detectable calcium transient on the first imaging day with isoflurane-anesthesia (90%, 124 cells, six FOVs) as well as on the first awake imaging day (93%, 234 cells, 10 FOVs; for criteria for detection of calcium transients, see Materials and Methods). Calcium transients occurred rather regularly in individual neurons in anesthetized mice. In contrast, amplitudes and durations of calcium transients were more heterogeneous in awake mice, including a substantial fraction of large and prolonged events (an example is colored in Fig. 1*E*). On average, calcium transients were smaller and of shorter duration during anesthesia compared with wakefulness (ΔF/F peak amplitude 45.0 ± 26.3% vs 89.5 ± 65.0%; FWHM 1.8 ± 2.3 vs 2.3 ± 2.1 s; mean ± SD, 2934 transients in 138 neurons for anesthetized and 2806 transients in 251 neurons for awake condition; *p* < 0.001 for both comparisons; two-sided Wilcoxon rank-sum test). These results indicate that CA3 pyramidal neurons show distinct patterns of neuronal activity in anesthetized compared with awake condition.

### Juxtacellular recordings of R-CaMP1.07-expressing CA3 pyramidal neurons

To relate R-CaMP1.07 calcium transients to AP patterns we performed acute experiments in anesthetized mice, obtaining simultaneous juxtacellular recordings and functional calcium imaging data from R-CaMP1.07-expressing CA3 pyramidal neurons (Fig. 2*A*,*B*). We extracted spike times using simple thresholding and temporally aligned calcium transients to the voltage recordings. Juxtacellular recordings revealed APs in variable numbers, often occurring in high-frequency bursts (Fig. 2*C*). The amplitude of consecutive spikes within a burst decreased over four to six APs, until no more spikes could be detected. For longer bursts, the AP amplitude often partially recovered after this initial decrease (Fig. 2*C*; Extended Data Fig. 2-1*A*,*B*). Burstiness was apparent in the bimodal distribution of ISIs, with two peaks at 5.3 ± 7.3 ms and 0.81 ± 1.51 s (mean ± SD), reflecting intraburst and interburst intervals, respectively (Extended Data Fig. 2-1*C*).

AP patterns in individual neurons correlated with the measured calcium transients (Fig. 2*C*). A spontaneous single AP-evoked ΔF/F transient on average had a peak amplitude of 11 ± 3% (*n* = 47 events, four neurons, three mice). With increasing number of APs, the ΔF/F amplitude of the corresponding calcium transients increased, following an approximately linear relationship up to 10 APs (Fig. 2*D*,*E*). The decay time constant of the evoked transient, as measured by an exponential fit, was around 0.3 s for single APs and remained <0.8 s for larger numbers of APs (Fig. 2*F*). These ground-truth data provide an important calibration resource that helps to interpret R-CaMP1.07 imaging data in CA3 neuronal populations more quantitatively.

### Large and prolonged calcium transients during wakefulness and locomotion

Taking advantage of this ground-truth calibration, we trained a supervised spike inference algorithm based on a deep neuronal network to temporally deconvolve ΔF/F transients and infer instantaneous SRs (Rupprecht et al., 2021; Materials and Methods). Deconvolution uncovered that during wakefulness, in contrast to anesthesia, calcium transients often were prolonged, indicating extended periods of spiking, sometimes over seconds (Fig. 3*A*). For quantification, we computed the mean ΔF/F value in a 3-s time window around the peak of a detected calcium transient (1 s before until 2 s after the peak), reflecting the integral cellular activity (overall number of APs) causing the calcium transient. The distribution of mean ΔF/F values was significantly shifted to higher values during wakefulness compared with anesthesia (23 ± 24% vs 11 ± 10%, mean ± SD, *p* < 0.001, two-sided Wilcoxon rank-sum test; Fig. 3*B*), in qualitative agreement with recent findings in CA1 (Yang et al., 2021). The distribution of mean ΔF/F values for the awake condition showed a pronounced tail of large events, with a substantial fraction reaching >100% mean ΔF/F. To account for these special events, we defined “awake large” events as those calcium transients with mean ΔF/F values larger than most anesthetized events (>95th percentile; Fig. 3*B*). According to this definition, 33.7% of all awake events were classified as large events. Overall, we classified our recorded calcium transients in “anesthetized” (*n* = 2934), “awake small” (*n* = 1859), and “awake large” (*n* = 947) events. We did not further divide calcium transients that were measured during anesthesia into small and large transients. The average shape and amplitude of anesthetized calcium transients resembled the small awake events, whereas the awake large events exhibited higher amplitudes and prolonged durations (Fig. 3*C*).

To further evaluate AP patterns that underlie detectable calcium transients as shown in Figure 3*A–C*, we performed additional analyses on the ground truth. For every transient detected according to our criteria, we analyzed the AP patterns in the 3-s window around the transient peak and compared it to AP firing patterns in time periods without detectable calcium transients. Detected calcium transients were induced by bursts of more than three APs (6.4 ± 4.4 APs without interruption; mean ± SD; bursts defined as APs with ISIs of <10 ms, according to the histogram in Extended Data Fig. 2-1*C*), indicating that our analysis misses a “floor” of single APs or very brief bursts that are hidden in the noise (we estimate this fraction could be as large as 30%). To permit a more direct interpretation of calcium signals in terms of underlying spikes, we generated a look-up table for the number of APs versus the mean ΔF/F value in the 3-s analysis window from the calcium transients detected in our ground-truth dataset (Fig. 3*B*, bottom). This relationship was approximately linear for small (<40%) mean ΔF/F values and tapered off at higher values. Note that this tapering-off corresponds to a supra-linear increase of mean ΔF/F values with the number of APs, possibly reflecting additional calcium influx caused by regenerative dendritic events associated with AP bursts (Grienberger et al., 2014; Raus Balind et al., 2019). Because large calcium transients with >40% mean ΔF/F were rare under anesthesia, this observation is based on only few data points, however, and therefore needs to be interpreted carefully. The variability of the estimated number of APs increased at high mean ΔF/F values, presumably indicating variations of the temporal profile of the underlying spike trains. Applying this look-up table to calcium transients measured during wakefulness, we estimate that small events reflect short bursts of APs or trains of up to 20 APs whereas the largest events with >100% mean ΔF/F presumably were caused by >30 APs within the 3-s window (Fig. 3*B*, top). As a limitation to this approach, it must be kept in mind that AP patterns, i.e., bursting versus continuous spiking, are not necessarily preserved between anesthetized and awake states.

The abundance of small calcium transients was comparable during anesthesia and awake resting condition but higher for awake running; moreover, large calcium transients occurred almost exclusively during running (Fig. 3*D*). Although the frequency distributions of small and large events across running speed were not significantly different (*p* = 0.64, Kolmogorov–Smirnov test), there was a trend for large events to particularly occur at the highest speeds (Fig. 3*E*). In summary, we observed especially large-amplitude calcium transients with prolonged duration during wakefulness, in particular during running.

### Stability and variability of neuronal activity and co-activity in CA3 across days

To assess how stable or variable the activity of CA3 pyramidal neurons is over days, we analyzed calcium transients measured repeatedly in the same neuronal populations over three consecutive days in both anesthetized and awake state (Fig. 4*A*,*B*). In the awake condition, 181 out of 251 neurons could be tracked across all 3 d (72%); in anesthesia sessions, 91 out of 138 neurons (66%) were consistently tracked. For each neuron, we calculated the mean ΔF/F peak amplitude, the average interevent interval (IEI) time and the average width (FWHM) for all calcium transients per day (Extended Data Fig. 4-1*A*,*C*). We quantified the stability of these features by correlating values recorded during one imaging day *N* + 1 with values for the same neurons from the previous day *N* (Lütcke et al., 2013; Fig. 4*C*). While the ΔF/F amplitude for the same neurons was relatively stable across days (Pearson’s correlation coefficient ρ = 0.50, 0.34, and 0.54 for anesthetized, awake resting, and awake running condition, respectively; all *p* < 6 × 10^−7^), correlation values were lower for FWHM (ρ = 0.20, 0.09, and 0.15, respectively; with *p* = 0.01, 0.23, 0.02) and IEI (ρ = 0.16, 0.04, and 0.17, respectively; with *p* = 0.09, 0.65, 0.04). Motivated by the observation that ΔF/F amplitudes were relatively stable, we specifically addressed the question how the distribution of large events (as defined in Fig. 3) changed over days across the population. In a subset of the neurons tracked across 3 d (47 out of 181 neurons) we observed large events on at least 1 d (33.7% of events in total). About a third of these neurons (33.4%) displayed large events on at least 2 d (chance level 18.2%; *p* = 0.012; Monte Carlo simulation of the null distribution) and a considerable fraction (14.9%) even on all three consecutive measurement days (chance level 0.85%; *p* < 1 × 10^−6^; Fig. 4*D*). These above-chance incidences indicate that a subset of neurons exists that is particularly prone to generate large events consistently over days.

Finally, we investigated CA3 neuronal activity on the population level. To assess synchrony of activity we calculated the percentage of co-active neurons per calcium transient per FOV, with co-activity defined as co-occurrence of calcium transients in a 1-s time window ranging from 0.5 s before to 0.5 s after an event (Materials and Methods; see also Extended Data Fig. 4-2*A–C* for variable time windows and transient peak time distribution of co-active transients). The percentage of co-active neurons per event was significantly higher than expected from chance level for all conditions (6.3 ± 3.6%, 14.1 ± 11.8%, and 12.4 ± 10.7% for anesthetized, awake running, and awake resting, respectively; mean ± SD; *n* = 2178, 6453, and 6282 calcium transient events, respectively; *p* < 1 × 10^−20^ for all conditions, corrected by subtracting shuffled data with randomized peak times; Wilcoxon signed-rank test; Fig. 4*E*). Additionally, we investigated the stability of co-active neuron pairs during wakefulness by comparing the percentage of coincident events within the 1-s window over two consecutive imaging days (Fig. 4*F*). Percentages ranged from 0% to 91.2% (10.5 ± 3.0%, mean ± SD) and remained relatively stable across two imaging days (Fig. 4*G*,*H*; Pearson’s correlation ρ = 0.34 compared with ρ = 0.06 for shuffled events; an even higher correlation value of ρ = 0.42 resulted from calculating co-activity based on estimated SRs; Extended Data Fig. 4-2*A*). For calcium transients recorded during anesthesia, the correlation of co-activity across days was somewhat lower (ρ = 0.12 compared with ρ = 0.05 for shuffled events; data not shown). To avoid potential confounds by signal contamination from neighboring neurons, we repeated this analysis after excluding nearest neighbors, yielding similar results (Extended Data Fig. 4-3). Together, these findings hint toward functional coupling of neuronal subpopulations in CA3 that is maintained across multiple days.

## Discussion

Our study contributes to the emerging field of *in vivo* calcium imaging of CA3 pyramidal neurons by establishing longitudinal imaging across days, comparing different behavioral states, and providing calibration in terms of spike patterns underlying the observed calcium transients. We found state-dependent neuronal responses with salient prolonged high-amplitude calcium transients in awake mice during locomotion. On the population level, we observed that during wakefulness individual calcium transients are embedded in surrounding network activity, with co-active neuron pairs maintaining their mutual co-activity over days.

Our juxtacellular recordings during anesthesia and the deconvolved calcium transients from awake imaging sessions indicate low mean firing rates but prominent burst events in CA3 pyramidal neurons, in line with previous studies (Henze et al., 2002; Frerking et al., 2005; Wittner and Miles, 2007; Mizuseki et al., 2012; Kowalski et al., 2016; Oliva et al., 2016; Ding et al., 2020). Compared with DG granule cells (Pilz et al., 2016) a much higher fraction of CA3 pyramidal neurons displayed clear calcium transients (>90% for all conditions in CA3; for comparison: <10% during anesthesia and around 50% during wakefulness in DG). The mean frequency of calcium transients across the entire population was 6- to 20-fold higher in CA3 than in DG, especially during anesthesia. Consistent with the high burstiness of CA3 pyramidal neurons, the vast majority of recorded calcium transients in our ground truth recordings reflected AP bursts rather than individual APs (84% of event-associated spikes were part of a burst of three or more spikes). The bimodal ISI distribution that we observed during anesthesia (Extended Data Fig. 2-1*C*) closely resembles previous results during light anesthesia (Kowalski et al., 2016) as well as during sleep and awake behavior (Frerking et al., 2005; Mizuseki et al., 2012). However, it is not straightforward to relate the changes in calcium transient frequency that we observed to changes in AP patterns. Moreover, recent *in vivo* whole-cell recordings found that theta oscillations were associated with membrane potential hyperpolarization in most CA3 pyramidal neurons (Malezieux et al., 2020), which could imply decreased average firing rates during running. However, theta periods included both resting and running periods and modulatory effects were quite heterogeneous across the CA3 population. Further investigations will be needed to clarify state-dependent modulation of membrane potential dynamics, AP patterns, and cellular calcium signals in CA3.

We estimate that the especially large and prolonged calcium events that we observed were caused by >30 APs over 3 s, indicating that a subset of CA3 pyramidal neurons can sustain firing rates of 10 Hz or higher during running. This spiking level is not too dissimilar from in-field firing rates observed in identified CA3 place cells (Mizuseki et al., 2012; Ding et al., 2020). As our experiments were conducted in the dark without salient spatial cues, we can only speculate that these events may relate to place cell or time cell properties. Rather than representing regular spiking, we interpret the large locomotion-related events as presumably reflecting a mixture of regular spikes and bursts at shortened interburst interval compared with resting conditions (note the “bumpy” SRs in the examples in Fig. 3*A*; see also Epsztein et al., 2011). Previous *in vivo* studies reported similarly long membrane potential depolarizations in medial entorhinal cortex during anesthesia (Hahn et al., 2012) and in CA1 during awake behavior, the latter termed “hippocampal motifs” that consisted of ∼2-s long AP sequences with above 5-Hz peak rate occurring during foraging behavior (Aghajan et al., 2015). These long-lasting bouts of activity in direct input and output regions of CA3 might be linked to the prolonged high amplitude calcium events that we observed. Further investigations are required in the future to resolve the electrophysiological basis of these special large events during awake running and their relationship to spatial navigation.

Another aspect that warrants further examination is the considerable heterogeneity in functional properties of CA3 pyramidal neurons that has been found along the proximo-distal axis. For example, input resistance and intrinsic excitability are higher in the proximal CA3 region compared with the distal region CA3a, where we performed our recordings (Sun et al., 2017, 2020). Additional factors contributing to the diversity include distinct synaptic inputs from medial entorhinal cortex (Fernandez-Lamo et al., 2019), prefrontal cortex (Rajasethupathy et al., 2015), and the supramammillary nucleus (Lu et al., 2015), as well as differences in dendritic length (Ding et al., 2020) and expression patterns of potassium and hyperpolarization-activated cyclic nucleotide–gated (HCN) channels (Sun et al., 2017; Raus Balind et al., 2019). Heterogeneity also exists along the dorsal-ventral axis (Sun et al., 2017) and between superficial and deep neurons within the pyramidal cell layer (Thompson et al., 2008; Cembrowski and Spruston, 2019). Future calcium imaging studies during awake behavior may help to link this neuronal diversity in CA3 to the propensity of cells to generate large calcium events as well as to specific behaviorally relevant population activity patterns.

Our juxtacellular recordings provide evidence that supra-linear calcium influx might occur with increasing AP numbers, suggesting additional sources that contribute to the mean ΔF/F values of large events (Fig. 3*D*). Additional calcium influx may have been caused by dendritic calcium spikes associated with complex spike bursts (Grienberger et al., 2014; Raus Balind et al., 2019), localized dendritic NMDA spikes (Brandalise et al., 2016), or dendritic plateau potentials induced by supra-linear integration of synaptic inputs (Takahashi and Magee, 2009). Plateau potentials and the associated complex spike bursts have been found to precede place field formation in CA1 neurons and may generally mediate behaviorally relevant plasticity in hippocampal pyramidal neurons (Bittner et al., 2015, 2017; Diamantaki et al., 2018).

The recurrent auto-associative nature of the CA3 network is suitable to support the formation of functional neuronal ensembles (Hopfield, 1982; Nakazawa et al., 2002; Guzman et al., 2016). In our experiments, neurons were more frequently co-active during wakefulness compared with anesthesia (Fig. 4*E*), hinting toward the recruitment of CA3 subpopulations during specific behavioral states or in particular sensory environments. Limited by the low temporal resolution of calcium imaging, we could not distinguish whether neurons were co-active on a synaptic time scale (milliseconds) or only on a longer time scale. Yet, we found that these co-active ensembles were relatively stable over consecutive days. Previous calcium imaging studies reported unstable space representations in place cells of CA1 and CA3 across days (Rubin et al., 2015; Hainmueller and Bartos, 2020), although some experiments indicate that representations can be stabilized (Kentros et al., 2004; Mankin et al., 2012; Julian et al., 2018). Despite unstable functional representations in single pyramidal neurons, neurons may maintain a stable affiliation to the same engram (Kinsky et al., 2018; Gonzalez et al., 2019) and spatial information could be stably encoded by whole-network activity patterns, based on pairwise co-activity (Stefanini et al., 2020). Across-day stability of a distributed engram, but variable activation patterns of the pyramidal neurons involved, may allow for flexible functional output of hippocampal subpopulations over time (Goode et al., 2020). In our experiments, a subpopulation of CA3 pyramidal neurons displayed large calcium events consistently across days. Furthermore, we found a subset of neurons that were stably co-active with other neurons within the same FOV across days. These results indicate at least some stability in the CA3 neuronal ensemble recruitment processes. Large calcium events associated with complex spike bursting might lead to plasticity in the recurrently connected CA3 network and could support the formation of functional engrams (Raus Balind et al., 2019). The emergence of co-active CA3 ensembles and their relevance for hippocampus-dependent behaviors warrant further investigations using longitudinal calcium imaging.

Because of the fundamental importance of the CA3 subfield in the cortico-hippocampal circuitry, we expect a surge of future *in vivo* CA3 imaging studies that will be facilitated by recent methodological advances. First, although two-photon imaging in DG and CA3 has been achieved with GCaMP indicators (Pilz et al., 2016; Hainmueller and Bartos, 2018), red-shifted calcium indicators may still be beneficial (Pilz et al., 2016; Kondo et al., 2017; Shemetov et al., 2021). Second, pushing excitation wavelengths further into the near-infrared wavelength is now possible with three-photon microscopy (Ouzounov et al., 2017), with entirely new opportunities for non-invasive hippocampus imaging through the neocortex (Ouzounov et al., 2017; Weisenburger et al., 2019). Finally, the combination of multi-photon imaging with optogenetic manipulation of specific neuronal ensembles, as recently demonstrated in the CA1 region (Robinson et al., 2020), will open new avenues for all-optical interrogation of hippocampal neuronal ensemble dynamics.

## Synthesis

Reviewing Editor: Liset Menendez de la Prida, Instituto Cajal CSIC Decisions are customarily a result of the Reviewing Editor and the peer reviewers coming together and discussing their recommendations until a consensus is reached. When revisions are invited, a fact-based synthesis statement explaining their decision and outlining what is needed to prepare a revision will be listed below. The following reviewer(s) agreed to reveal their identity: Christophe Mulle, Tristan Shuman.

Your ms was evaluated by two reviewers and myself. We all agree there is value in your data. While your main findings are not entirely novel, we very much appreciate imaging from CA3 cells and simulatenous in vivo juxtacellular and GCaMP imaging data. HOwever, we also have a number of concerns you may need to carefully address before a final recommendation can be made for publication.

On the methodological side we have the following major concerns:

1. You use manually selected ROIs to quantify calcium signals. This could lead to spurious correlations between neurons based on neuropil contamination or cross-talk between nearby neurons. Using demixing methods (e.g., CNMF) or at least quantifying the relationship between neuron distance and co-activity would mitigate this and make the co-activity findings more convincing. This may be relevant for the interpretation of large events.

2. You made the commendable effort to compare the firing rate of spikes recorded in the juxtacellular mode to the amplitude of Ca2+ transients. We feel you should include a more thorough analysis indicating what firing properties trigger what will eventually be defined as a detectable Ca2+ transient. What is described is merely a number of spikes in a quite broad (∼3s) window, and does not clearly differentiate between a series of individual spikes at sustained frequencies (e.g 1 to 5 Hz) and bursts of spikes. Bursts are not clearly defined: 3+ spikes occurring with ∼6ms inter-spike-intervals? Similarly, what firing patterns elicit no detectable Ca2+ transient and what percentage of spikes are missed entirely? This will help the authors make more accurate statements regarding the presumed firing rate based on Ca2+ activity. There is some switching back and forth between assuming the transient rate corresponds to the firing rate or the rate of bursts (lines 315-17 vs 367-8, 371-3). These two interpretations can have different functional consequences.

3. There is mention of using a neural network to predict firing rates from dF/F traces, but it is unclear in the paper whether this is used in any subsequent analysis. Figure 3A shows the deconvolved firing rates, but the accuracy of the algorithm is impossible to determine from the data shown. We would like you to provide a measure of the accuracy of the neural network’s output based on the ground truth dataset.

4-Figure 2 is entirely devoted to another method of inferring spikes from Ca2+ transients which is also unclear when and where it is used for subsequent analysis. Figure 2E and 3B (bottom panel) appear to be showing similar information (peak vs mean dF/F): are they in fact based on the same data? We need these issues to be addressed and clarified.

Regarding data results and interpretation:

5-Presumably you are targeting CA3a, which is borderline with CA2 and the region is very heterogenous. We would like to know how specific the Girk4-cre to fully exclude this confounding. Importantly, there are proximodistal differences along CA3 in terms of intrinsic properties and input integration (especially from MEC), plus neuronal heterogeneity across deep/superficial layers. We feel this should be somehow considered. We wonder whether part of the variability (e.g. % or location of cells expressing large event) may be explained by some of these differences.

6- We also feel that comparison with existing extracellular data should be taken carefully. As the authors say in the text, Oliva et al., 2016 found increased firing rates, but Mizuseki et al., 2012 found reduced burst rates during running (however, the Mizuseki study compared running with sleep, rather than running with quiet wakefulness, as is done in the current study, so the results are difficult to compare). Similarly, Mizuseki and Oliva data have different biases along the proximodistal axis (only Oliva 2016 considers the proximodistal/deep/superficial dimension). The extracellular nature of these data may be also considered, while in vivo intracelular recordings may provide a complementary view. Moreover, if it is true that Ca2+ imaging in this study misses most if not all spikes occurring outside bursts (due to the 20% threshold described in line 194), we would argue that one should refrain from making statements about the presumed firing rate of cells. In addition -although you made the point- all interpretation about firing rates in the awake mice should be taken with caution because the spike/Ca2+ transient relationship may well be very different in awake conditions.

7. There is considerable mention of the supralinear relationship between number of spikes and Ca2+ transients, but this is not well-shown in any figure or analysis. The methods point out this is shown in 3B (bottom); We recommend to include a linear reference line in the figure.

8. We contend that it is difficult to establish that the relationship is truly supralinear, since there are so few datapoints with mean dF/F values >50%. What is the precedence for using the mean dF/F measure (methods explained in lines 277-8), rather than the peak or the area under the curve? There is a comparison with a Yang et al., 2020 (line 282) that would presumably provide some clarification, but the full citation is not included in the list of references.

9. It is true that the amount of coactivity and the stability across days is higher than that in a shuffled dataset. However, because of the slow sampling rate and the broad duration of the window (3 seconds, within the range of duration of a theta bout), it is quite difficult to extract important information on these “co-active neurons”. And it is certainly risky to relate this co-activity with connected CA3 neurons. It is also difficult to make claims about the level of stability since there are no other conditions, behaviors, or brain regions for comparison. Related to this point, it is unclear whether the blue dots in Figure 4H are awake, anesthetized, or both. Maybe a comparison of the rho values between the two conditions would give more insight.

10.Fig 4E - this finding is pretty clear but a formal statistical test would be helpful

11. Please, add estimation statistics whenever possible (e.g. Fig.2E,F and 3D)

Minor comments to be addressed

1. Line 186-8 states: “We determined the peak amplitude of the recorded calcium transient and counted the number of underlying APs (burst events within APs maximally spread over a 200-ms period were included in this analysis).” Please clarify the part in parentheses.

2. Since some of the main claims of the paper are centered around stability across days, please include a measure of what percentage of cells can be reliably tracked across days. If a similar measure exists in the literature, please reference it for comparison.

3. In a related point, it seems that a larger proportion of cells can be tracked across days in the awake condition compared with anesthetized (91/382 for anesthetized vs 182/388 for awake). If this calculation is correct, do you have a hypothesis for the apparent disparity? Can this be merely linked to higher general frequency of transients in awake vs. anesthetized mice?

4. Line 260-3 gives the mean and median of the distribution shown in Extended Data Fig. 2-1 C. I would suggest that since the distribution is clearly bimodal, the mean and median of the two peaks can be calculated separately, rather than reporting the mean and median of the entire distribution.

5. It is perhaps not correct to refer to Mizuseki et al., 2012 to make claims about firing rates of CA3 pyramidal cells in running vs quiet wakefulness periods (Lines 317 and 378), since the comparisons made by Mizuseki were between running and sleep states. See also our points 5 and 6 above

6. Please provide more details about the shuffling procedures used for Fig 4F and 4H. Were the dF/F traces time-shifted with one another or were pseudo-random times selected for surrogate Ca2+ transients? How many times was the shuffle performed?

7. For Figures 4F-J, do the results qualitatively change if the deconvolved firing rate traces (from Figure 3A) are used instead of the dF/F traces? The advantage here would be to also decrease the time window for coincidence below 3s, as the functional relevance of such a wide window is difficult to determine.

8. Line 367-8 states: “Consistent with the high burstiness of CA3 pyramidal neurons, the vast majority of recorded calcium transients reflected AP bursts rather than individual APs.” It is not clear to me that this analysis has actually been done (see major point #1), but it could be performed on the juxtacellular dataset.

9. Line 371-3 states: “Our data are consistent with state-dependent modulation of AP patterns, with temporally more dispersed APs and reduced burst propensity during locomotion (Mizuseki et al., 2012).” This claim does not appear to be backed up by the analyses they performed and should be clarified.

10. Line 405-6 states: “In our experiments, neurons were more frequently co-active during wakefulness compared to anesthesia (Fig. 4G)...” However, it does not appear that the statistics have been reported to support this statement. If cells are more active, isn’t it expected to have more co-active cells, with the time window used?

11. Line 417-9 states: “In our experiments, a subpopulation of CA3 pyramidal neurons displayed large calcium events consistently across days and showed stable co-activity with other neurons of the same FOV.” This statement makes it sound like the same group of cells display both properties, but it is not clear that the analysis was performed this way. Please clarify.

12. Figure 3D shows the rate of transients over different states. However, from this figure, it is unclear how many cells are contributing to the changes. As suggested above, a more useful measure would be the transient rate per cell, rather than lumped over the entire population.

13. Figure 3E is not easy to interpret since there is no information about how much time is spent at each speed. I would suggest that the y-axis should be a frequency or rate rather than a normalized count.

14. Figure 4 C and D (left) appear to have the same data. D is easy to read and interpret but C less so; I would suggest that panel C is not necessary.

15. The legend for Figure 4F does not seem to match the description in the text (Lines 338-41). Specifically, it is unclear whether one data point in the figure is an entire recording session of one FOV, or a single transient.

## Author Response

Response to reviewer comments

General remarks:

We thank the reviewers and the editor for their valuable comments and suggestions to

improve the manuscript. We have now revised the manuscript and incorporated their

feedback. Specifically, we have performed additional analyses to provide a more

detailed description of the relationship of cell-attached recorded spikes and

simultaneously measured calcium signals, and we extended our population analysis to

deconvolved calcium transients (estimated spike rates). Furthermore, various

clarifications were incorporated into the main text.

Please find below our point-by-point responses.

Synthesis of Reviews:

Computational Neuroscience Model Code Accessibility Comments for Author

(Required): Not a computational paper. However it uses some codes for analysis

multicellular GCaMP data.

Significance Statement Comments for Author (Required):

This paper reports data from simultaneous in vivo juxtacellular and calcium imaging in

CA3 and describes basic firing patterns in both awake and anesthetized mice.

Comments on the Visual Abstract for Author (Required):

A visual abstract should be provided

We now provide a visual abstract for our paper.

Synthesis Statement for Author (Required):

Your ms was evaluated by two reviewers and myself. We all agree there is value in your

data. While your main findings are not entirely novel, we very much appreciate imaging

from CA3 cells and simulatenous in vivo juxtacellular and GCaMP imaging data.

HOwever, we also have a number of concerns you may need to carefully address

before a final recommendation can be made for publication.

Thank you for appreciating our efforts to advance CA3 calcium imaging and to provide

juxtacellular ground truth data.

Responses to the major concerns on the methodological side:

1. You use manually selected ROIs to quantify calcium signals. This could lead to

spurious correlations between neurons based on neuropil contamination or cross-talk

between nearby neurons. Using demixing methods (e.g., CNMF) or at least quantifying

the relationship between neuron distance and co-activity would mitigate this and make

the co-activity findings more convincing. This may be relevant for the interpretation of

large events.2

Thank you for this valuable suggestion. We agree with the reviewers’ concern that it is

necessary to assess potential contamination of neuronal calcium signals by surrounding

neuropil or neighboring neurons, in particular for the analysis of neuronal co-activity.

As suggested, we compared our manual ROI selection with source extraction using

CNMF on multiple exemplary sessions in both awake and anesthetized conditions.

ROIs detected by CNMF overlapped with our manually selected ROIs by 79 {plus minus}10%

(Extra Figure 1A). Distances between centroids showed a similar distribution for both

methods (Extra Figure 1B; 30.0 19.8 um and 31.6 19.6 um, respectively; mean

s.d.; p=0.99, Kolmogorov-Smirnov test). However, neurons that were easily identified by

their morphology by eye, but did not show obvious calcium transients during a recording

session, were not detected by CNMF. Furthermore, false positive ROIs had to be

removed manually after CNMF detection. From our experience, using CNMF often

requires such manual clean-up or manual pre-seeding of ROIs. Thus, for the rather

small number of neurons in our FOVs, we preferred manual selection of ROIs chosen in

a rather conservative way, avoiding the bias of CNMF to detect only neurons with high

activity levels, which could have affected our results especially regarding comparison of

activity levels across days.

Extra Figure 1. Comparison of ROI selection. A, Color-coded ROIs detected by CNMF (left), with manual

selection (middle), and overlaid (right). B, Distribution of pairwise ROI distances for CNMF and manual

selection. C, Decontamination of ΔF/F signals using FISSA. Manually selected ROIs of five neighbouring

neurons (left) and respective unaltered and FISSA-corrected ΔF/F traces (right).3

To assess in how far contamination of F/F signals by neuropil and neighboring

neurons is a problem in our dataset, we applied the FISSA toolbox (Keemink et al.,

Scientific Reports 2018, 8:3493), which supposedly performs better than CNMF for

neuropil correction. We evaluated the F/F traces from multiple exemplary sessions of

neighboring neurons before and after correction (Extra Figure 1C). Against our

expectations, FISSA correction seemed to increase noise levels and also introduce

more contamination between neurons (e.g. between neuron 3 and 4). Based on these

findings of insufficient F/F correction with FISSA and upon further close visual

inspection of the raw videos, we convinced ourselves that the quality of our data is

sufficiently high (also given the rather large signals) to produce reliable calcium traces.

The example traces in Extra Figure 1C demonstrate how signals in neighboring neurons

(e.g. neurons 2 and 3) are clearly distinct. We also convinced ourselves that the large

events observed arise from genuine single-neuron activity patterns and were not

caused by the merged activity of multiple overlapping somas. We therefore chose to

refrain from any extra preprocessing of the F/F traces.

Finally, we followed your suggestion and quantified the dependence of pairwise co activity on neuronal distance, using the manually selected ROIs. This new analysis is

shown in the new Extended Data Fig. 4-3. Only for the smallest distances (<10 um),

with neurons nearly touching each other, we found that mean Pearson’s correlation

values were clearly higher compared to farther apart neuron pairs. This result hints

towards cross-neuronal contamination at these shortest distances (Extended Data

Figure 4-3A). To verify our findings, we therefore restricted the analysis of co-activity

across days to neuron pairs that were at least 10 μm apart from each other. The results

of this restricted analysis were comparable to the results obtained for all neuron pairs

and the main effect, that co-activity patterns were relatively stable across days, was still

present (Extended Data Fig. 4-3B). We conclude that our statements regarding stable

co-activity of neuron pairs are not confounded by cross-neuronal contamination.

2. You made the commendable effort to compare the firing rate of spikes recorded in

the juxtacellular mode to the amplitude of Ca2+ transients. We feel you should include a

more thorough analysis indicating what firing properties trigger what will eventually be

defined as a detectable Ca2+ transient. What is described is merely a number of spikes

in a quite broad (∼3s) window, and does not clearly differentiate between a series of

individual spikes at sustained frequencies (e.g 1 to 5 Hz) and bursts of spikes. Bursts

are not clearly defined: 3+ spikes occurring with ∼6ms inter-spike-intervals? Similarly,

what firing patterns elicit no detectable Ca2+ transient and what percentage of spikes

are missed entirely? This will help the authors make more accurate statements

regarding the presumed firing rate based on Ca2+ activity. There is some switching

back and forth between assuming the transient rate corresponds to the firing rate or the

rate of bursts (lines 315-17 vs 367-8, 371-3). These two interpretations can have

different functional consequences.4

We have now included a more thorough analysis - based on our ground truth

recordings - of what spike patterns most likely underlie the detected calcium transients.

First, we like to point out that we used a conservative detection criterion for calcium

transients in the analysis of population imaging data (threshold crossing of 3 s.d. of the

ΔF/F trace for anesthetized data; see Methods). This conservative criterion was

necessary due to the relatively low signal to noise-ratio (SNR) of the in vivo population

imaging data during wakefulness. We now applied the same detection criterion to

calcium transients in the ground truth data set (as before in Fig. 3B, bottom) and

analyzed the firing patterns underlying these detected calcium transients. As discussed

in more detail below, all ground truth data were recorded during anesthesia. Also note

that the SNR of the ground truth data was slightly higher because FOVs were imaged

zoomed-in on the recorded neuron.

First, we inspected the time windows around detected calcium transients (between -1

and +2 s of a detected F/F peak) and analyzed whether spikes fell into any of those

windows. Overall, 70.6% of ground truth spikes lay within these windows (‘event associated spikes’), indicating that ∼30% of spikes were missed entirely by our analysis

(‘not event-associated spikes’). This relatively high percentage reflects the conservative

criterion that we chose for calcium event detection during awake recordings.

Next, to understand the temporal distribution of spikes underlying detected events, we

used the peak of a detected calcium transient from ground truth data and aligned the

spikes within each transient to the respective peaks, resulting in a peri-event histogram

of spikes. This plot, which we now show in the new panel of Extended Data Fig. 2-1D,

clearly displays an increased spike rate just prior to a transient peak.

Finally, we explored the firing patterns during time windows around detected calcium

transients by analyzing the bursts. Following the distribution of inter-spike intervals in

Extended Data Fig. 2-1C, we used an ISI of 10 ms as a cutoff to define bursts. On

average, a randomly selected ‘event-associated spike’ was part of a burst of 6.4 {plus minus} 4.4

APs (mean {plus minus} s.d.), while ‘not event-associated spikes’ were on average part of a burst

of 3.6 {plus minus} 2.3 APs. We conclude that bursts of >3 APs were required to elicit calcium

transients that could be detected based on our criterion. We have amended the

description of this analysis in the Methods and added these numbers to a paragraph in

the Results section (lines 325ff).

Admittedly, uncertainty about the interpretation of results thus remains, in part also

because it has not been examined how well the analyses of firing patterns under

anesthesia translate to awake conditions (although AP-evoked calcium transients are

expected to be fairly similar; see for example Greenberg et al., Nat Neurosci 2008,

11:749). In the discussion, we compare the firing patterns from ground truth recordings

during anesthesia to existing electrophysiological recordings of bursts in CA3 (previous

lines 367-8 and 371-3, as indicated in your comment). More importantly, we also

discuss how the awake large events might be different from events during anesthesia.

One indication comes from the shape of the ΔF/F transients during anesthesia and 5

wakefulness and from the respective deconvolved firing rates (Fig. 3A). The shapes of

the transients suggest that firing patterns underlying awake events are not single

discrete bursts (which would result in a sharp large transient with rapid decay as for

anesthetized transients) but a temporally more distributed firing pattern (which might

well include also bursts with variable number of APs). We write in the discussion (lines

438ff):

"Previous in vivo studies reported similarly long membrane potential depolarizations in

medial enthorinal cortex during anesthesia (Hahn et al., 2012) and in CA1 during awake

behaviour, the latter termed “hippocampal motifs” that consisted of ∼2-s long AP

sequences with above 5 Hz peak rate occurring during foraging behavior (Aghajan et al.,

2015). These long-lasting bouts of activity in direct input and output regions of CA3

might be linked to the prolonged high amplitude calcium events that we observed.”

As we write in the same context, further investigations will be required to understand the

true firing patterns underlying large awake events.

We believe that both aspects, the comparison of ground truth firing patterns under

anesthesia with the literature, and the finding of temporally more dispersed firing

patterns, based on Fig. 3A, should be mentioned. This might appear like switching forth

and back, but it reflects the uncertainty about the true firing patterns that underlie awake

calcium transients.

3. There is mention of using a neural network to predict firing rates from dF/F traces, but

it is unclear in the paper whether this is used in any subsequent analysis. Figure 3A

shows the deconvolved firing rates, but the accuracy of the algorithm is impossible to

determine from the data shown. We would like you to provide a measure of the

accuracy of the neural network’s output based on the ground truth dataset.

In the original manuscript we indeed used deconvolved data only for Fig. 3A.

The accuracy of the algorithm has been quantified in a separate paper, to which we

refer in the text and which is available as bioRxiv preprint (Rupprecht et al., bioRxiv,

2021; https://doi.org/10.1101/2020.08.31.272450). This paper introduces this new

algorithm based on neural networks in detail and provides a full characterization.

In the revised manuscript, we now report the overall accuracy metrics for the noise

conditions and frame rates that we used in our study. The metrics were obtained by

training the network on all but one neuron of the ground truth and applying it to the

remaining data (‘leave-one-out strategy’). By temporally smoothing the ground truth

used for training, one can choose the temporal resolution achieved by the network. Very

high target temporal resolution would result in lower overall accuracy of predictions.

Given the relatively low SNR of our data, we therefore chose a relatively low temporal

resolution (smoothing with a Gaussian with about 470-ms width). All this information is

now provided in a new full paragraph in the Methods section (lines 192ff):6

"We also utilized the ground truth data set (n = 4 neurons from 3 mice; a total of 33 min of

recording and 5025 APs) to train a supervised algorithm based on neural networks to

deconvolve calcium transients and estimate the underlying spike rates. The deconvolution

algorithm, which we present in detail in a separate paper (Rupprecht et al., 2020), was

trained on the R-CaMP1.07 ground truth data, which were re-sampled to the 10-Hz frame

rate used for awake imaging. The noise level of the ground truth data was adjusted to

match the noise level of each neuron of the population imaging data by adding Poisson

noise. Spike trains used to train the network were temporally filtered with a Gaussian

(∼470 ms full-width-half-maximum, FWHM). Prediction of spike rates using this approach

is expected to show correlation values of 0.79 0.16 (mean s.d.) with the ground truth

data, thus explaining about 60% of the variance (Rupprecht et al., 2020).”

Triggered by your comment, we now used deconvolved traces for additional analyses.

First, we show the mean spike rate estimated by deconvolution underlying the detected

events in the ground truth dataset (Extended Data Fig. 2-1D). Second, we included a

panel showing the estimated spike rates underlying calcium transients in co-active

neurons (Extended Data Fig. 4-2B), supporting our new choice of a 1-s window for the

analysis of co-activity. Third, we found that the main findings of stable co-activity in

neuron pairs was also recovered when analyzing deconvolved traces (see our response

to minor point #7 below). Fourth, we included the co-activity analysis based on spike

rate estimates in analyses described below under point (9) (Extended Data Figs. 4-2A

and 4-3B).

4-Figure 2 is entirely devoted to another method of inferring spikes from Ca2+ transients

which is also unclear when and where it is used for subsequent analysis. Figure 2E and

3B (bottom panel) appear to be showing similar information (peak vs mean dF/F): are

they in fact based on the same data? We need these issues to be addressed and

clarified.

In Figure 2, we use the standard characterization of calcium indicators by analyzing how

calcium transient amplitude (and decay time) depend on the number of APs in short

time windows (here 200 ms; AP sequences in such window are therefore often loosely

described as bursts). These plots help to evaluate the sensitivity of a calcium indicator

and the relationship of the number of near-synchronously elicited APs and the evoked

calcium transient amplitude (see for example Lütcke et al., 2013, Helmchen et al.,

Biophys J 1996, 70:1069; Chen et al., Nature 2013, 499:295). Such characterization

obviously requires simultaneous electrophysiological ground-truth data. It helps to

estimate the amplitude and temporal shape of the single-AP evoked calcium transient

and to assess how non-linearly the calcium indicator behaves.

Figure 3B (bottom) is an analysis that is indeed based on the same data, but it is more

tailored towards our analysis of detected calcium events. It addresses the question:

When we detect a calcium event with a given mean ΔF/F, what is the number of

underlying spikes? This is possible because the mean ΔF/F value is an integral 7

measure that for a linear indicator should be approximately proportional to the total

number of APs in the chosen window.

We understand that these similar analyses might lead to some confusion. In the revised

manuscript, we have rewritten the Data Analysis section in the Methods (lines 185ff)

and modified text in the Results section (lines 325ff) to clarify as best as we could, in

which way the two analyses are different and which separate questions they allowed us

to address.

Responses to comments regarding data results and interpretation

5-Presumably you are targeting CA3a, which is borderline with CA2 and the region is

very heterogeneous. We would like to know how specific the Girk4-cre to fully exclude

this confounding. Importantly, there are proximo-distal differences along CA3 in terms of

intrinsic properties and input integration (especially from MEC), plus neuronal

heterogeneity across deep/superficial layers. We feel this should be somehow

considered. We wonder whether part of the variability (e.g. % or location of cells

expressing large event) may be explained by some of these differences.

To the best of our knowledge our imaging planes indeed were positioned in distal CA3

(CA3a), as these neurons were the first labelled cells that became visible when we

focused down from the corpus callosum. We assume to have recorded deep as well

superficial pyramidal neurons in the same FOV as our imaging plane provided a cross section through the stratum pyramidale. As far as we know from the literature, starting

with the original publication introducing the Grik4-Cre mouse line (Nakazawa et al.

2002), expression in this driver line is rather specific to the CA3 region (see for example

the recent paper El-Gaby et al., Nat Neurosci 2021, 24(5):694). This is also consistent

with our own histology (Fig. 1). Nonetheless, the transition zone between CA3 and CA2,

whether it is sharp or more gradient-like, indeed still warrants further characterization.

To indicate some caution, we now write in the Methods that this mouse line shows

"expression of Cre-recombinase rather specific to CA3 hippocampal pyramidal neurons"

(line 117).

Unfortunately, we lack large morphological 3D two-photon image stacks spanning the

entire CA3, neither did we attempt to identify the recorded neurons post-mortem (which

is possible but difficult). Therefore, we feel that we cannot assign the recorded neurons

to specific positions along the CA3 axes with sufficiently high precision to consider it in

our analysis or make any statement about heterogeneity. We do agree that this

heterogeneity is an important aspect and that in the future population calcium imaging

should be beneficial for a more detailed analysis of the relationship of functional

properties and anatomical position of CA3 neurons (on all three axis). In the revised

manuscript we added a discussion of these aspects, mentioning possible sources of

variability among pyramidal neurons, in the Discussion section (lines 447ff):8

Another aspect that warrants further examination is the considerable heterogeneity in

functional properties of CA3 pyramidal neurons that has been found along the proximodistal axis. For example, input resistance and intrinsic excitability are higher in the

proximal CA3 region compared to the distal region CA3a, where we performed our

recordings (Sun et al., 2017; Sun et al., 2020). Additional factors contributing to the

diversity include distinct synaptic inputs from medial entorhinal cortex (Fernandez-Lamo et

al., 2019), prefrontal cortex (Rajasethupathy et al, 2015) and the supramammiliary

nucleus (Lu et al., 2015), as well as differences in dendritic length (Ding et al., 2020) and

expression patterns of potassium and HNC channels (Sun et al., 2017; Raus Balind et al.,

2019). Heterogeneity also exists along the dorsal-ventral axis (Sun et al., 2017) and

between superficial and deep neurons within the pyramidal cell layer (Thompson et al.,

2008; Cembrowski and Spruston, 2019. Future calcium imaging studies during awake

behavior may help to link this neuronal diversity in CA3 to the propensity of cells to

generate large calcium events as well as to specific behaviorally relevant population

activity patterns.

6- We also feel that comparison with existing extracellular data should be taken

carefully. As the authors say in the text, Oliva et al., 2016 found increased firing rates,

but Mizuseki et al., 2012 found reduced burst rates during running (however, the

Mizuseki study compared running with sleep, rather than running with quiet wakefulness,

as is done in the current study, so the results are difficult to compare). Similarly,

Mizuseki and Oliva data have different biases along the proximodistal axis (only Oliva

2016 considers the proximodistal/deep/superficial dimension). The extracellular nature

of these data may be also considered, while in vivo intracelular recordings may provide

a complementary view. Moreover, if it is true that Ca2+ imaging in this study misses

most if not all spikes occurring outside bursts (due to the 20% threshold described in

line 194), we would argue that one should refrain from making statements about the

presumed firing rate of cells. In addition -although you made the point- all interpretation

about firing rates in the awake mice should be taken with caution because the

spike/Ca2+ transient relationship may well be very different in awake conditions.

Thank you for pointing out the problems of interpreting the Oliva et al. and Mizuseki et al.

studies, in particular when comparing with our calcium imaging data. We have now

either removed or rewritten the respective sentences in the main text (e.g. lines 420ff).

In our response to your major point (2) (see above) we have described what information

we could extract regarding the firing patterns underlying detected calcium events and

the percentage of event-associated vs. not-event-associated spikes.

We agree that one should be careful when making statements about firing rates based

on calcium imaging, especially for neurons that show bursting behavior. We therefore

carefully checked and adjusted the wording of the relevant sentences and particularly

tried to avoid any confusion between “firing rates” and “calcium event rate”.

7. There is considerable mention of the supralinear relationship between number of 9

spikes and Ca2+ transients, but this is not well-shown in any figure or analysis. The

methods point out this is shown in 3B (bottom); We recommend to include a linear

reference line in the figure.

We added a linear reference line to Fig. 3B (fit to small events with mean ΔF/F < 40%).

In addition, we added a model-free fit of all data points together with a 95% confidence

corridor in the bottom plot of Figure 3B.

In principle, a supralinear relationship between detected calcium responses and the

underlying number of APs may reflect either the non-linearity of the indicator itself or

additional calcium influx at high activity levels. We interpret our data as a clear

indication of the latter scenario for the following reasons. Although most commonly used

genetically encoded calcium indicators show cooperative binding of calcium ions, which

leads to a non-linear response behavior in the concentration range around their Kd, the

red calcium indicator R-CaMP1.07 used in our study approximates more linear behavior

than the widely used green GCaMP6f (Hill coefficients of 1.7 0.1 and 2.1 {plus minus} 0.1,

respectively; Inoue et al., 2015, 12:64). Consistent with previous data (Bethge et al.,

2017), R-CaMP1.07 therefore shows a relative linear dependence of calcium transients

amplitude on number of APs (see Fig. 2E). The alternative cause of the supra-linear

response is additional calcium influx, for example caused by regenerative potentials in

dendrites or through other mechanisms (e.g. calcium release). This increased calcium

influx is then reported by the indicator. We argue that the supralinear responses in our

data are consistent with this second scenario, which is also in line with the previous

reports of additional activation of calcium currents due to dendritic regenerative

potentials associated with strong AP bursts (see also lines 335ff and 460ff).

8. We contend that it is difficult to establish that the relationship is truly supralinear,

since there are so few datapoints with mean dF/F values >50%. What is the precedence

for using the mean dF/F measure (methods explained in lines 277-8), rather than the

peak or the area under the curve?

In our ground truth data set, data points for calcium transients with a mean ΔF/F

values >40% are indeed less frequent as for smaller transients. However, we would still

argue that these data points give evidence for a supra-linear relationship of mean F/F

values vs. AP number, indicating additional calcium sources as for example dendritic

regenerative events. To better visualize this effect, we added a linear reference line and

performed a fit of the data using Gaussian process regression to establish 95%

confidence intervals (Figure 3B). For values >45% F/F, all data points without

exception are clearly supra-linear. As a note of caution, we have added the following

sentence in the Results section (lines 340ff):

"Because large calcium transients with >40% mean ΔF/F were rare under anaesthesia,

this observation is based on only few data points, however, and therefore needs to be

interpreted carefully.”10

The mean ΔF/F measure is actually equivalent to area-under-the curve, as it

corresponds to the integral in the 3-s time window divided by 3-s. We chose this integral

measure of activity, rather than amplitude, because the shape of the detected calcium

events indicated that they are triggered not only by simple phasic spiking events (e.g.

bursts) but by spike patterns and multiple bursts spread over time (see our comment in

lines 307ff). Hence, we argue that an area-based measure provides a better estimate of

underlying spiking activity, as it approximately reflects the total number of APs in the

chosen time window.

There is a comparison with a Yang et al., 2020 (line 282) that would presumably provide

some clarification, but the full citation is not included in the list of references.

The reference for Yang et al., is now incorporated into the reference list. We apologize

for the confusion generated by the omission. This study is, however, focused on

studying differences of firing properties in CA1 neurons between awake and

anesthetized conditions using extracellular recordings and two-photon imaging. It is not

related to the observed supra-linearity. Yang et al. assess the effect of different types of

commonly used general anesthetics and they report reduced spiking of units and

decorrelated activity of ensembles under anesthesia. We have now better clarified that

we meant to indicate that their observation of higher spiking activity during wakefulness

is in qualitative agreement with the distinct activity distributions for anesthetized and

awake condition (Fig. 3B) that we found in CA3 neurons (lines 312ff).

9. It is true that the amount of coactivity and the stability across days is higher than that

in a shuffled dataset. However, because of the slow sampling rate and the broad

duration of the window (3 seconds, within the range of duration of a theta bout), it is

quite difficult to extract important information on these “co-active neurons”. And it is

certainly risky to relate this co-activity with connected CA3 neurons.

Thank you for your feedback. We agree that a 3-s time window provided only a coarse

measure of co-activity and might have biased our population analysis to higher coactivity values. Given the limited temporal resolution of calcium imaging, we certainly

cannot make statements about co-activity (synchronicity) on the short time scale of

synaptic interactions. We also agree that there is no direct relation between a coarse

measure of co-activity and anatomical connectivity. Despite these caveats, we believe

that also weak evidence about co-active neurons can be informative when interpreted

carefully.

To address the concerns of overestimating the number of co-active neurons in our data

set as far as possible - given limitations of sampling rate and signal-to-noise levels - we

followed two approaches:

First, we now used a shorter time window of 1 s for the co-activity analysis (-0.5 s

before to 0.5 after detected peaks). We settled on this duration as a result of a

systematic analysis of how window size affected our analysis of co-stability across days. 11

This extra analysis is now shown in the new Extended Data Figure 4-2A, where we

analyzed Pearson correlation of co-activity across days for window sizes between 0.1

and 4.5 s for awake and anesthetized data (and additionally for awake deconvolved

calcium transients). For the 0.1 s window the analysis was performed on the single

imaging frame for which the transient peak of the event was detected. All other windows

started 0.5 s before the transient peak and varied in their duration as indicated. Time

windows longer than 0.7 s created similar Pearson’s correlation values whereas

correlation dropped for shorter windows (to 0.23 for an 0.5-s window and to 0.04 for

single-frame analysis). Based on this outcome, we decided to use the 1-s time window,

which however admittedly still is a coarse measure of co-activity.

Second, we investigated the shape and peak position of co-active calcium transients (as

well as estimated spike rates, SR) aligned to the peak of the originally detected

reference event (Extended Data Fig. 4-2B,C). We found that the average calcium

transient in the co-active CA3 neuron subpopulation peaked in the same imaging frame

as the reference calcium transient. The average SR trace peaked 200 ms before the

calcium transient peak for short events and 300 ms for large events. Co-active calcium

transients peaked predominantly in the same time frame as the reference event (34% of

all events within the 1-s window for small events, 27% for large events; Extended Data

Fig. 4-2C). Together, these analyses show that a significant fraction of events are likely

to be co-active with a temporal precision even higher than our newly chosen 1-s window.

We have adapted the text in the Methods (lines 225ff) and Results (lines 381ff) sections,

to make these points clearer. In addition, we now explicitly mention the limitation of our

co-activity analysis in the discussion (lines 473ff):

"Limited by the low temporal resolution of calcium imaging, we could not distinguish

whether neurons were co-active on a synaptic time scale (milliseconds) or only on a

longer time scale.”

It is also difficult to make claims about the level of stability since there are no other

conditions, behaviors, or brain regions for comparison. Related to this point, it is unclear

whether the blue dots in Figure 4H are awake, anesthetized, or both. Maybe a

comparison of the rho values between the two conditions would give more insight.

The blue data points in previous Figure 4H were based on awake population recordings.

The label in the new version of this panel (Figure 4G, based on using a 1-s time window

for analysis) is now more precise (“Awake”). To analyze the stability across days more

systematically, we now provide the Pearson correlation values for different correlation

time windows in Extended Data Fig. 4-2A. For the newly chosen 1-s window, correlation

values were ρ(awake)=0.34 (compared to 0.06 shuffled) and ρ(anesth.) = 0.12 (0.05

shuffled), indicating a stronger correlation between cell pairs across days for the awake

condition than in anesthetized data. We added the co-activity Pearson correlation

values for the anesthetized condition to Extended Data Fig. 4-2A and the comparison

between awake and anesthetized condition is now also included in the Results section

(lines 394ff).12

While we agree that the claims that can be made based on our data are limited (and we

have tried to avoid any overstatements), we hope that these modifications have

strengthened our analysis. Further investigations will be required to assess stability of

co-activity at higher temporal resolution.

10.Fig 4E - this finding is pretty clear but a formal statistical test would be helpful

We used Monte-Carlo simulations to determine the percentage of neurons showing

large events that would be observed over 2 or 3 days by chance. We repeated

simulations 5x106

times and used the resulting distribution of percentages across

repetitions to determine the p-value of the experimental value with respect to the

random null distribution. The chance level was found to be 18.2% and 0.85% (p = 0.012

and p < 10-6

), for the case of events across 2 and 3 days, respectively.

11. Please, add estimation statistics whenever possible (e.g. Fig.2E, F and 3D)

We agree with you that estimation statistics based on confidence intervals and effect

sizes are useful tools that should replace hypothesis testing whenever possible and

applicable.

In Fig. 2E,F, the data presentation is, however, not focused on measured effect sizes

but on a pure description of our findings. We therefore find that the methods applied are

appropriate (plot of central value with a measure of the spread, Fig. 2E, or uncertainty,

Fig. 2F).

Regarding the statistics related to Fig. 3D, we now mention the size of the observed

effects in addition to p-values by using Gaussian propagation of the standard error of

the means. Gaussian error propagation enables us to calculate ratios between different

conditions while taking the uncertainty into account.

Response to reviewers’ minor concerns

1. Line 186-8 states: “We determined the peak amplitude of the recorded calcium

transient and counted the number of underlying APs (burst events within APs maximally

spread over a 200-ms period were included in this analysis).” Please clarify the part in

parentheses.

This analysis procedure only refers to Figure 2 and is the standard way to evaluate the

dependence of calcium indicator signal amplitude on number of evoked APs (see also

our response to major methods concerns, point #4). For a linear indicator (neglecting

saturation) the individual transients just add up and thus result in linear summation of

calcium transients if APs occur in a time window that is short relative to the decay time

of the indicator. To approximate such a condition, one usually defines a relatively short 13

time window, within which variable numbers of APs have to occur. Similar to previous

studies, we here chose a 200-ms window, so that APs had to occur in relatively rapid

succession. This is the reason why we called them ‘burst events’ but we realize that this

has been confusing with the definition of natural bursts in the typical CA3 pyramidal

neuron firing patterns (as a note aside: many of the events used for Fig. 2D indeed were

bursts). We hope that this explanation clarifies the part in parentheses.

To avoid confusion, we have now expanded the explanatory part in the Methods section

(lines 187ff) and do not refer to ‘burst events’ anymore:

"We determined the peak amplitude of isolated calcium transients (i.e., with no spiking

activity in a 2-s period prior to the first AP associated with the calcium transient) and

counted the number of underlying APs. To focus on quasi impulse-like responses, we

only considered transients with APs that occurred within a time window of maximally

200-ms duration. For averaging, calcium transients were aligned to the first AP of a

given event.”

2. Since some of the main claims of the paper are centered around stability across days,

please include a measure of what percentage of cells can be reliably tracked across

days. If a similar measure exists in the literature, please reference it for comparison.

Please see our response to the next point.

3. In a related point, it seems that a larger proportion of cells can be tracked across

days in the awake condition compared with anesthetized (91/382 for anesthetized vs

182/388 for awake). If this calculation is correct, do you have a hypothesis for the

apparent disparity? Can this be merely linked to higher general frequency of transients

in awake vs. anesthetized mice?

Thank you for pointing out this inconsistency, which was caused by an unfortunate error

in calculating the total number of imaged neurons. We have now corrected this error

throughout the manuscript. Using the correct numbers, we found that in the awake and

anesthetized conditions only 43 and 31 neurons, respectively, were lost over three days.

This is equivalent to a rate of losing 1.35 and 1.33 neurons, respectively, per FOV and

imaging day. Moreover, with the correct numbers, there is no difference between awake

and anesthetized conditions. Thus, the different firing properties of neurons under

awake and anesthetized conditions have no detectable effect on our ability to track a

neuron over days. Please find a more detailed account of neurons numbers in the table

below:

awake anesthetized

Corrected number of cells 251 138

Number of cells tracked over 3 days 181 (72%) 91 (66%)14

Cells recorded on 1-2 days 70 (28%) 47 (34%)

We now report the percentage of cells tracked over 3 days in the main text (line 359ff).

A more detailed overview across all cells and recording days, that went into the

longitudinal analysis of various calcium transient properties, is provided in Extended

Data Figure 4-1A-C.

Longitudinal calcium imaging studies in CA3 are rare and currently we are aware of only

one study (Hainmüller and Bartos, 2018) that tracks the same CA3 pyramidal neurons

over two days. However, this study only reports the number of neurons which showed

place field properties on one day but it does not state the total number of neurons or the

percentage of neurons that could be tracked across days.

4. Line 260-3 gives the mean and median of the distribution shown in Extended Data

Fig. 2-1 C. I would suggest that since the distribution is clearly bimodal, the mean and

median of the two peaks can be calculated separately, rather than reporting the mean

and median of the entire distribution.

Based on the reviewers’ suggestions we split our observations in logarithmic time units

into two clusters using k-means clustering. The first cluster was centered around a

mean {plus minus} s.d. of 5.3 {plus minus} 7.3 ms (median 4.7 ms), while the second cluster was centered

around a mean of 0.81 s (s.d. 1.51 s; median: 0.89 s). We now report the means {plus minus} s.d.

for both peaks in the main text (lines 292ff) and the legend for Fig. 2-1.

5. It is perhaps not correct to refer to Mizuseki et al., 2012 to make claims about firing

rates of CA3 pyramidal cells in running vs quiet wakefulness periods (Lines 317 and

378), since the comparisons made by Mizuseki were between running and sleep states.

See also our points 5 and 6 above

We agree with your point of view and have changed the main text accordingly. Please

see our response to points 5 and 6 above.

6. Please provide more details about the shuffling procedures used for Fig 4F and 4H.

Were the dF/F traces time-shifted with one another or were pseudo-random times

selected for surrogate Ca2+ transients? How many times was the shuffle performed?

Because our definition of co-activity is based on assessing the co-occurrence of

detected calcium transient peaks within a short time window, we simply used random

shuffling of peak times and assessed the mean of the resulting co-activity for 100

repeats. We have clarified the shuffling approach in the Methods section (lines 230ff).

7. For Figures 4F-J, do the results qualitatively change if the deconvolved firing rate

traces (from Figure 3A) are used instead of the dF/F traces? The advantage here would 15

be to also decrease the time window for coincidence below 3s, as the functional

relevance of such a wide window is difficult to determine.

As suggested, we now also performed the neuron-pair co-activity analysis using the

deconvolved calcium traces. To this end, we performed peak detection on the estimated

spike rate (SR) traces (see Methods) and used the detected SR events and their peak

times as the basis for this alternative co-activity analysis. As shown in the Extra Figure 2

below, the results are highly compatible with the analysis based on F/F traces,

showing again a significantly higher fraction of co-active neurons in the same FOV

compared to shuffled controls and high Pearson correlation for the co-activity

comparison across days, with ρ(SR)= 0.42 even higher compared to ρ(ΔF/F) = 0.34.

The latter effect is also seen in the comparison of Pearson correlation values for

variable time window sizes (Extended Data Figure 4-2A), where SR-based values are

larger compared to F/F based values for window sizes of 1 s, but become more similar

for long time windows.

Because the alternative analysis using SR traces instead of F/F traces did not

qualitatively change our results, we decided not to include Extra Figure 2 in the revised

manuscript. However, if you feel that we should add it, we would be happy to include

the extra plots.16

Extra Figure 2. Comparison of co-activity based on calcium transients and estimated spike rates. A,

Percentage of neurons in a FOV that show co-activity within a 1-s time window surrounding a detected

event, based on either F/F traces or estimated spike rate (SR) traces (p < 1×10-20 for all event types;

two-sided Wilcoxon signed-rank test; ***p<0.001; box displays median and 25th and 75 percentiles,

whiskers indicate 1

st and 99th percentile; resting vs. running: 1.20 {plus minus} 0.02; 0.97 {plus minus} 0.01, ratio of means {plus minus}

error propagation of SEM for ΔF/F and estimated SR, respectively). B, Comparison of percentage of coactive events per neuron pair over consecutive imaging days, based on either F/F traces or estimated

spike rate (SR) traces (ΔF/F data: ρ = 0.34, shuffled ρ = 0.06; estimated SR data: ρ = 0.42, shuffled ρ =

0.06; left plot is identical to Fig. 4G). C, Normalized 2D density plot as in Fig. 4H but based on the SR plot

in panel B, right.

8. Line 367-8 states: “Consistent with the high burstiness of CA3 pyramidal neurons, the

vast majority of recorded calcium transients reflected AP bursts rather than individual

APs.” It is not clear to me that this analysis has actually been done (see major point #1),

but it could be performed on the juxtacellular dataset.

In our response to major point 2 (see above) we discuss in more detail how detected

calcium transients are related to AP bursts in the ground truth data set with juxtacellular

recordings.

To more directly address your specific question: we find that 84% of event-associated

spikes are part of a burst of 3 or more spikes. Here, we use the criterion introduced in 17

our reply to major point 2 that two spikes are part of a burst if the inter-spike interval

between subsequent spikes is {less than or equal to}10 ms.

9. Line 371-3 states: “Our data are consistent with state-dependent modulation of AP

patterns, with temporally more dispersed APs and reduced burst propensity during

locomotion (Mizuseki et al., 2012).” This claim does not appear to be backed up by the

analyses they performed and should be clarified.

We agree with your assessment that the connection to the Mizuseki paper in this

context does not add substantial value. We have therefore removed this sentence.

10. Line 405-6 states: “In our experiments, neurons were more frequently co-active

during wakefulness compared to anesthesia (Fig. 4G)...” However, it does not appear

that the statistics have been reported to support this statement. If cells are more active,

isn’t it expected to have more co-active cells, with the time window used?

Following the advice of reviewers, we shortened the time window for analysis to 1 s to

reduce possible spurious co-activity due to higher firing rates. If neurons are generally

more active in a given condition, this could indeed lead to higher levels of co-activity.

However, this effect also would be reflected in the shuffled data as mean event rates

are conserved. To allow for a comparison between the different conditions we now

correct each condition by subtracting the mean of the respective shuffled control and

report the ratio between the conditions using the corrected values. Furthermore, we

performed Gaussian error propagation of SEM of the ratios. The corresponding

sentence in the Results now reads (line 385ff):

"(Fig. 4E; 6.3 {plus minus} 3.6%, 14.1 {plus minus} 11.8% and 12.4 {plus minus} 10.7% for anesthetized, awake running,

and awake resting, respectively; mean s.d.; n = 2178, 6453 and 6282 calcium transient

events, respectively; p < 1×10-20 for all conditions, corrected by subtracting shuffled data

with randomized peak times; Wilcoxon signed-rank test).”

And in the legend to Fig. 4E:

"Percentage of neurons in a FOV that show co-activity within a 1-s time window surrounding a

detected event, compared to randomly shuffled event times (***p<0.001; box displays median and

25th and 75th percentiles, whiskers indicate 1st and 99th percentiles; resting vs. anesth. 2.15 {plus minus} 0.05,

running vs. anesth. 2.57 {plus minus} 0.06, resting vs. running 1.20 {plus minus} 0.02; ratio of means {plus minus} error propagation

of SEM).”

11. Line 417-9 states: “In our experiments, a subpopulation of CA3 pyramidal neurons

displayed large calcium events consistently across days and showed stable co-activity

with other neurons of the same FOV.” This statement makes it sound like the same

group of cells display both properties, but it is not clear that the analysis was performed

this way. Please clarify.18

Thank you for pointing out this misleading wording. We did not intend to indicate that

these two separate effects were found for the same subpopulation. We have now

clarified the sentence as follows (lines 485ff):

"In our experiments, a subpopulation of CA3 pyramidal neurons displayed large calcium

events consistently across days. Furthermore, we found a subset of neurons that were

stably co-active with other neurons within the same FOV across days.”

12. Figure 3D shows the rate of transients over different states. However, from this

figure, it is unclear how many cells are contributing to the changes. As suggested above,

a more useful measure would be the transient rate per cell, rather than lumped over the

entire population.

The plots indeed show mean frequency of calcium transients per neuron and session.

This is now clarified in the main text (line 386) and figure legend.

13. Figure 3E is not easy to interpret since there is no information about how much time

is spent at each speed. I would suggest that the y-axis should be a frequency or rate

rather than a normalized count.

To address this comment, we updated Fig. 3E and now present the frequency of small

and large events as a function of run speed.

14. Figure 4 C and D (left) appear to have the same data. D is easy to read and

interpret but C less so; I would suggest that panel C is not necessary.

We now moved former Fig. 4C to Extended Data Fig. 4-1. We still would like to keep it

in the manuscript because it offers additional information (visual comparison across

three subsequent days instead of the more abstract plots of day N+1 vs. day N).

15. The legend for Figure 4F does not seem to match the description in the text (Lines

338-41). Specifically, it is unclear whether one data point in the figure is an entire

recording session of one FOV, or a single transient.

The plot in Figure 4 (now panel E) is based on data points that represent the

percentage of co-active neurons in the same FOV for each detected calcium transient.

We have now better clarified this in the plot and the legend.
